# Multivariate indicators of disease severity in COVID-19

**DOI:** 10.1038/s41598-023-31683-9

**Published:** 2023-03-29

**Authors:** Joe Bean, Leticia Kuri-Cervantes, Michael Pennella, Michael R. Betts, Nuala J. Meyer, Wail M. Hassan

**Affiliations:** 1 Department of Biomedical Sciences, School of Medicine, University of Missouri – Kansas City, 2411 Holmes Street, Kansas City, MO 64108 USA; 2 Department of Microbiology, Perelman School of Medicine, University of Pennsylvania, Philadelphia, PA 19104 USA; 3 Institute for Immunology, Perelman School of Medicine, University of Pennsylvania, Philadelphia, PA 19104 USA; 4 Division of Pulmonary, Allergy and Critical Care, Department of Medicine, Center for Translational Lung Biology, Lung Biology Institute, Perelman School of Medicine, University of Pennsylvania, Philadelphia, PA 19104 USA

**Keywords:** Immunology, Viral infection

## Abstract

The novel coronavirus pandemic continues to cause significant morbidity and mortality around the world. Diverse clinical presentations prompted numerous attempts to predict disease severity to improve care and patient outcomes. Equally important is understanding the mechanisms underlying such divergent disease outcomes. Multivariate modeling was used here to define the most distinctive features that separate COVID-19 from healthy controls and severe from moderate disease. Using discriminant analysis and binary logistic regression models we could distinguish between severe disease, moderate disease, and control with rates of correct classifications ranging from 71 to 100%. The distinction of severe and moderate disease was most reliant on the depletion of natural killer cells and activated class-switched memory B cells, increased frequency of neutrophils, and decreased expression of the activation marker HLA-DR on monocytes in patients with severe disease. An increased frequency of activated class-switched memory B cells and activated neutrophils was seen in moderate compared to severe disease and control. Our results suggest that natural killer cells, activated class-switched memory B cells, and activated neutrophils are important for protection against severe disease. We show that binary logistic regression was superior to discriminant analysis by attaining higher rates of correct classification based on immune profiles. We discuss the utility of these multivariate techniques in biomedical sciences, contrast their mathematical basis and limitations, and propose strategies to overcome such limitations.

## Introduction

As of the date of this writing, the novel coronavirus SARS-CoV-2—the causative agent of the novel coronavirus disease (COVID-19)—has sickened over 0.67 billion people and resulted in more than 6.8 million deaths around the globe^1^. The clinical presentation varies widely, ranging from an asymptomatic infection to a severe viral pneumonia, which can rapidly progress to acute respiratory distress syndrome (ARDS) and multi-organ failure^2,3^. Identifying reliable early predictive markers of severe and critical disease and deciphering the underlying mechanisms responsible for such divergent disease outcomes are urgently needed.

Mild to moderate COVID-19 disease is characterized by upper respiratory tract symptoms (e.g., cough, sore throat), fever, headache, and mild pneumonia (< 50% lung involvement); severe disease is defined by > 50% lung involvement, dyspnea, and hypoxia in addition to any combination of the symptoms of mild/moderate disease; and critical disease is characterized by respiratory failure, shock, and multi-organ system dysfunction. Most symptomatic patients (81%) experience mild or moderate disease, while 14% and 6% experience severe and critical illness, respectively^4^. In patients who develop severe disease, the median time from the onset of symptoms to the development of ARDS is 8–12 days^4^. This delay before the onset of life-threatening complications is an opportunity for clinicians to detect high-risk patients to intervene and potentially curb mortality.

Many predictors of disease progression have been identified. The CDC defines certain groups who are at increased risk for severe infection and possibly death, including older adults and patients with specific comorbidities, including cancer, chronic kidney disease, liver disease, chronic lung disease, diabetes mellitus, and immune suppression, among other comorbidities^5^. The use of clinical calculators based on predictive algorithms has helped enable early detection of high-risk patients and allowed clinicians to focus their attention and triage resources. These clinical calculators use patients’ vital signs, simple laboratory values, and comorbidities to predict clinical course and mortality^6^. Overall, they have shown good negative predictive value for mortality^6,7^. However, the success of such calculators in predicting severe disease is relatively low, with sensitivity for four of the most popular calculators ranging from 23.8 to 84.2%, and specificity ranging from 35.9 to 69.0%^6^. Clearly, the main value of these calculators is in their clinical application rather than in uncovering the underlying mechanisms of disease. Immune profiles have the potential to provide novel insights into the underlying mechanisms, as well as serving as biomarkers for clinical applications. One of the most comprehensive immune profiling studies in COVID-19 patients is that of Kuri-Cervantes et al.^8^, which we have selected to reanalyze using our multivariate modeling methods hoping to better define the most useful biomarker profiles for predicting disease severity and shed some new light on the pathophysiology of severe versus mild and moderate disease.

It has been realized that the nature of patient’s own immune responses likely plays a major role in the pathophysiology of COVID-19. Several groups have attempted to characterize the differences in immune responses between the various disease severity groups and have discovered several significant trends. These studies tend to analyze either the cytological response to infection, often using mass or flow cytometry, or the levels of cytokines and other plasma proteins^8–13^. A limited number of these studies have attempted to develop models to predict clinical progression based upon immunological profiling early in infection. Several groups have found that patients with COVID-19 do not share a single common immunotype, but rather fall into one of a number of immunotypes that correlate with clinical presentation. Most commonly, three separate immunotypes have been identified: an appropriate immune response associated with lower risk of mortality, an excessive immune response, and an inadequate or low immune response^14–16^. Individuals demonstrating excessive or inadequate immunotypes on admission tend to deteriorate clinically and develop more severe disease^14–16^. Groups have analyzed various factors to assess the immune response to COVID-19 infection, including cytokines and other soluble serum factors^14–18^, changes in cell populations^9,10,16–18^, and gene expression changes in immune cells^16,17^. Some of these groups primarily analyzed factors which distinguish severe COVID-19 patients from healthy controls^9,17,18^, some have compared factors which differentiate severities of infection and anticipate clinical progression^9,16–19^, and still others have produced models which predict clinical prognosis/severity based upon initial immunological data by defining distinct ‘immunotypes’ of infection^10,14,15^.

Kuri-Cervantes’ study identified several features of COVID-19 including leukocytosis accompanied by expansions of both neutrophil and eosinophil populations^8^. Severe COVID-19 disease has been correlated with CD4^+^ and CD8^+^ T-cell decline and an increased neutrophil-to-lymphocyte ratio^8,20^. Further, a decrease in the dendritic cell population and an increase in the monocyte population have also been observed^8^. Some of the most interesting and significant changes observed in severe COVID-19 patients occur in the lymphocyte populations. Overall, a lymphopenia is typically observed, driven most heavily by decreased T cell populations. This includes a decrease in the frequency of the CD4^+^ T cells, CD8^+^ T cells, NK cells, and CD8^+^ mucosal associated invariant T cells (CD8^+^ MAIT), seen primarily in individuals with severe disease^8,21^. More detailed analysis show that this decrease is not seen in CD4^+^ or CD8^+^ memory T cells^8^. This decrease in lymphocytes, combined with the increase in neutrophils, contributes to a proposed independent risk factor: the neutrophil-to-lymphocyte ratio, or alternatively the neutrophil-to-T-cell ratio, whose increase has been correlated with severe disease^8,22^. The overall B cell population also demonstrates a decrease in severe patients^21^, but a consistent and interesting finding in patients with severe disease is a substantial increase in the plasmablast population^8^. More detailed analysis shows that this expansion is oligoclonal, with a few clones contributing to the majority of the circulating plasmablast population in patients with severe disease. This oligoclonality is stronger with severe disease than moderate disease or recovered patients. Analysis of antibody characteristics demonstrates elongation of CDR3 sequences, which has been hypothesized to contribute to pathogenesis by producing multi-reactive/nonspecific antibodies^8^. Beyond mere changes in cell populations, activation of lymphocytes has also been shown to be altered by severe COVID-19 infection. Increased activation of CD4^+^ memory T cells and CD8^+^ MAIT has been described^8^. It should be noted that despite these general trends in response to severe COVID-19 infection, the immunological response has been shown to be very heterogeneous. as discussed previously, some groups have proposed different immunotypes and some have correlated these immunotypes with different clinical outcomes^10,14,15^.

The main goals of the current study is to evaluate the predictive power of the immunological variables tested in Kuri-Cervantes et al.^8^ in identifying COVID-19 severity groups, identify the most distinguishing features of each severity group, compare these features to existing literature, and clearly present the reader with a discussion on the validity and limitations of statistical methods used here and elsewhere. We employed discriminant analysis (DA) and binary logistic regression to reanalyze the work presented by Kuri-Cervantes et al.^8^. Two main objectives motivated this work, the first of which is to identify combination of features that is most effective in distinguishing between either COVID-19 patients and normal control or the various disease severity groups among COVID-19 patients. The second objective is to determine the relative importance of each of these features for group discrimination. Using principal component analysis (PCA), Kuri-Cervantes et al. identified T cell activation in CD4^+^ and CD8^+^ memory T cells, frequency of plasmablasts, and frequency of neutrophils as the top parameters associated with severe COVID-19^8^. Although PCA has the advantage of unbiased exploration of data partitioning, DA directly addresses group discrimination. Both techniques combine correlated variables into eigenvectors, called principal components in PCA and canonical discriminant functions in DA. A key difference, however, is that PCA selects the vectors that maximize the amount of variance explained, while DA maximizes group discrimination. Binary logistic regression is also designed to directly address group separation. We also present an evaluation of the significance of the contribution of each of the variables and models used in the study, and thereby assisting the reader in perceiving the appropriate level of confidence through which the data should be viewed.

## Results

### Evaluation of the fitness of data for discriminant analysis

Most of our predictor variables deviated, sometimes substantially, from normal Gaussian distribution. Univariate normality of each variable (v = 171) was tested in four datasets: healthy controls, moderate COVID-19, severe COVID-19, and combined moderate/severe COVID-19. Therefore, there were 684 variable/dataset combinations (V_i_). Normal distribution was indicated by a Shapiro–Wilk’s *W* statistic equal to, or approaching, “1” and a *p*-value greater than 0.05. V_i_s that did not significantly deviate from normality using a *p*-value cutoff of 0.05 [245 (35.8%)] showed *W* values ranging from 0.812 to 0.990. Skewness (a measure of distribution asymmetry around the mean) values for these V_i_s were mostly within the − 1-to-1 range, except for 58 V_i_s (23.7%); none of the latter V_i_s, however, fell outside the − 2-to-2 range. For kurtosis (a measure of tailedness or clustering of datapoints in tails as opposed to the peak of the distribution curve), 109 V_i_s (44.5%) were outside the − 1-to-1 range, of which 20 (8.2%) were also outside the − 2-to-2 range. The remainder (55.5%) were within the − 1-to-1 range (Supplemental Fig. S1, Table S1). This means, using a − 1-to-1 cutoff, skewness and kurtosis agreed with Shapiro–Wilk’s test *p*-value 76.3% and 55.5% of the time, respectively. Among the 439 V_i_s (64.2%) that significantly deviated from normality according to the Shapiro–Wilk’s test, 49 (11.2%) and 90 (20.5%), respectively, showed skewness and kurtosis values within the − 1-to-1 range. Therefore, among these V_i_s, skewness and kurtosis data agreed with Shapiro–Wilk’s test results 88.8% and 79.5% of the time, respectively. These V_i_s showed *W* values ranging from 0.273 to 0.920. (Supplemental Table S1). Overall, Shapiro–Wilk’s test showed 84.4% and 70.9% agreement with skewness and kurtosis data, respectively, using a cutoff range of − 1-to-1 for the latter two.

The absence of multicollinearity was confirmed using correlation matrices generated using Pearson moment correlation coefficient. There is no precise consensus on the correlation coefficient threshold above which multicollinearity is presumed to exist. Thresholds as low as 0.40 and as high as 0.85 have been reported^23^, but the most commonly used threshold in our experience ranges from 0.70^23^ to 0.80^24^. In this study, we used 0.8 as our threshold and we found that CD69^+^, CXCR5^+^, CD38^+^, HLA-DR^+^, CD38^+^ HLA-DR^+^, Ki67^+^, and PD1^+^ subsets of total memory CD4^+^ T cells were often highly correlated with cell populations carrying the same surface markers among central, effector, and transitional memory CD4^+^ T cells. Total memory CD8^+^ T cells expressing these same surface markers were often highly correlated with cell populations carrying the same markers among central, effector, effector CD45RA^+^, and transitional memory CD8^+^ T cells. CD69^+^, CXCR5^+^, CD38^+^, HLA-DR^+^, CD38^+^ HLA-DR^+^, Ki67^+^, and PD1^+^ subsets of total memory CD8^+^ T cells were also highly correlated with multiple other cell populations including subsets of CD4^+^ T cells (Supplemental Tables S2–S4).

Non-parametric Levene’s test showed that most of the variables in each of our 3 models were homoscedastic with *p*-values greater than 0.05. Thirteen, 29, and 11 of the 171 variables used in each model showed *p*-values less than 0.05, suggesting heteroscedasticity among these variables in models 1, 2, and 3, respectively (Supplemental Table S5). Box’s M test was also performed yielding *p*-values of 5.7528 × 10^−14^ and 9.8324 × 10^−22^ for models 1 and 2, respectively (Table 1). Box’s M test could not be calculated in SPSS for Model 3 for technical reasons. As discussed in more detail in the "Discussion" section, the non-parametric Levene’s test is more reliable in assessing homoscedasticity in non-normal data^25,26^. We also evaluated outliers in our datasets. Using the criteria described in the “Methods” section, we identified 52, 31, 134, and 216 outliers in the control, moderate, severe, and COVID-19 groups, respectively. Out of 171 variables, there were 45, 31, 79, and 94 variables containing 1 or more outliers in the control, moderate, severe, and COVID-19 groups, respectively (Supplemental Tables S6–S10).

**Table 1:** Evaluation of the fitness of the discriminant models and the relative importance of canonical functions within each model.

Model 1								
Suitability of data for discriminant analysis								
Pooled within-groups matrices (correlation between predictor variables)								Box’s M test *p-*value (cutoff > 0.001)
Pearson correlations	NK cells		ActSMB	T cells		ActNeut	ImGran	5.7528 × 10^−14^
NK cells	1							
ActSMB	− 0.192		1					
T cells	− 0.080		0.144	1				
ActNeut	0.055		− 0.534	0.117		1		
ImGran	0.377		− 0.339	0.120		0.184	1	

Model fitness								
Step	Wilks’ λ				*p-*value			
1	0.351				1.1055 × 10^−8^			
2	0.189				1.0226 × 10^−11^			
3	0.125				3.3432 × 10^−13^			
4	0.083				1.261 × 10^−14^			
5	0.065				6.6843 × 10^−15^			
Model	0.065				4.8677 × 10^−15^			

Discriminant function discriminatory power								
	Eigenvalue			% Variance			Canonical correlation	
First discriminant function	4.155			67.7			0.898	
Second discriminant function	1.985			32.3			0.815	

Model 2								
Suitability of data for discriminant analysis								
Pooled within-groups matrices (correlation between predictor variables)								Box’s M test *p-*value (cutoff > 0.001)
Pearson correlations	MAIT	En38NK		ActNB		En27NK	Temra	9.8324 × 10^−22^
MAIT	1							
En38NK	0.147	1						
ActNB	− 0.226	− 0.254		1				
En27NK	0.192	− 0.202		0.297		1		
Temra	− 0.093	0.068		− 0.039		− 0.343	1	

Model fitness								
Step	Wilks’ λ				*p-*value			
1	0.459				1.4335 × 10^−7^			
2	0.347				8.8178 × 10^−9^			
3	0.224				3.6834 × 10^−11^			
4	0.191				1.9134 × 10^−11^			
5	0.166				1.3565 × 10^−11^			
Model	0.166				1.101 × 10^−11^			

Discriminant function discriminatory power								
	Eigenvalue			% Variance			Canonical correlation	
First discriminant function	5.033			100.0			0.913	

Model 3								
Suitability of data for discriminant analysis								
Pooled within-groups matrices (correlation between predictor variables)								Box’s M Test *p-*value (cutoff > 0.001)
Pearson correlations	Monocyte HLA-DR MFI	ActSMB	NK cells	CXCR5^+^CD8^+^ MAIT	cDC HLA-DR MFI	CD45RA^+^ effector memory CD8^+^ T cells	Neutrophils	N/A
Monocyte HLA-DR MFI	1							
ActSMB	− 0.501	1						
NK cells	0.231	− 0.333	1					
CXCR5^+^CD8^+^ MAIT	− 0.094	− 0.252	− 0.224	1				
cDC HLA-DR MFI	0.097	− 0.165	− 0.158	− 0.227	1			
CD45RA^+^ effector memory CD8^+^ T cells	− 0.053	− 0.022	0.207	0.374	0.156	1		
Neutrophils	− 0.163	− 0.007	− 0.228	0.251	0.012	− 0.397	1	

Model fitness								
Step	Wilks’ λ				*p-*value			
1	0.413				3 × 10^−6^			
2	0.196				3.1293 × 10^−9^			
3	0.0.142				6.324 × 10^−10^			
4	0.113				4.3273 × 10^−10^			
5	0.095				4.9018 × 10^−10^			
6	0.072				2.1319 × 10^−10^			
7	0.058				2.0125 × 10^−11^			
Model	0.058				8.7331 × 10^−11^			

Discriminant function discriminatory power								
	Eigenvalue			% Variance			Canonical correlation	
First discriminant function	16.219			100.0			0.971	

Model variables were sequentially incorporated into the model, one at a time, and are listed in the order they were incorporated.

*NK cells* natural killer cells, *en27NK* CD27^+^ NK cells, *en38NK* CD38^+^ NK cells, *actNeut* activated HLA-DR^+^ neutrophils, *imGran* Ki67^+^ immature granulocytes, *Temra* CD27^−^CD45RA^+^ effector memory CD8^+^ T cells, *actNB* CD21^+^CD27^−^Ki67^+^ B cells, *actSMB* CD21^−^CD27^+^CD38^lo^ B cells.

### Construction and evaluation of discriminant models tailored for specific clinical applications

**Figure 1:**
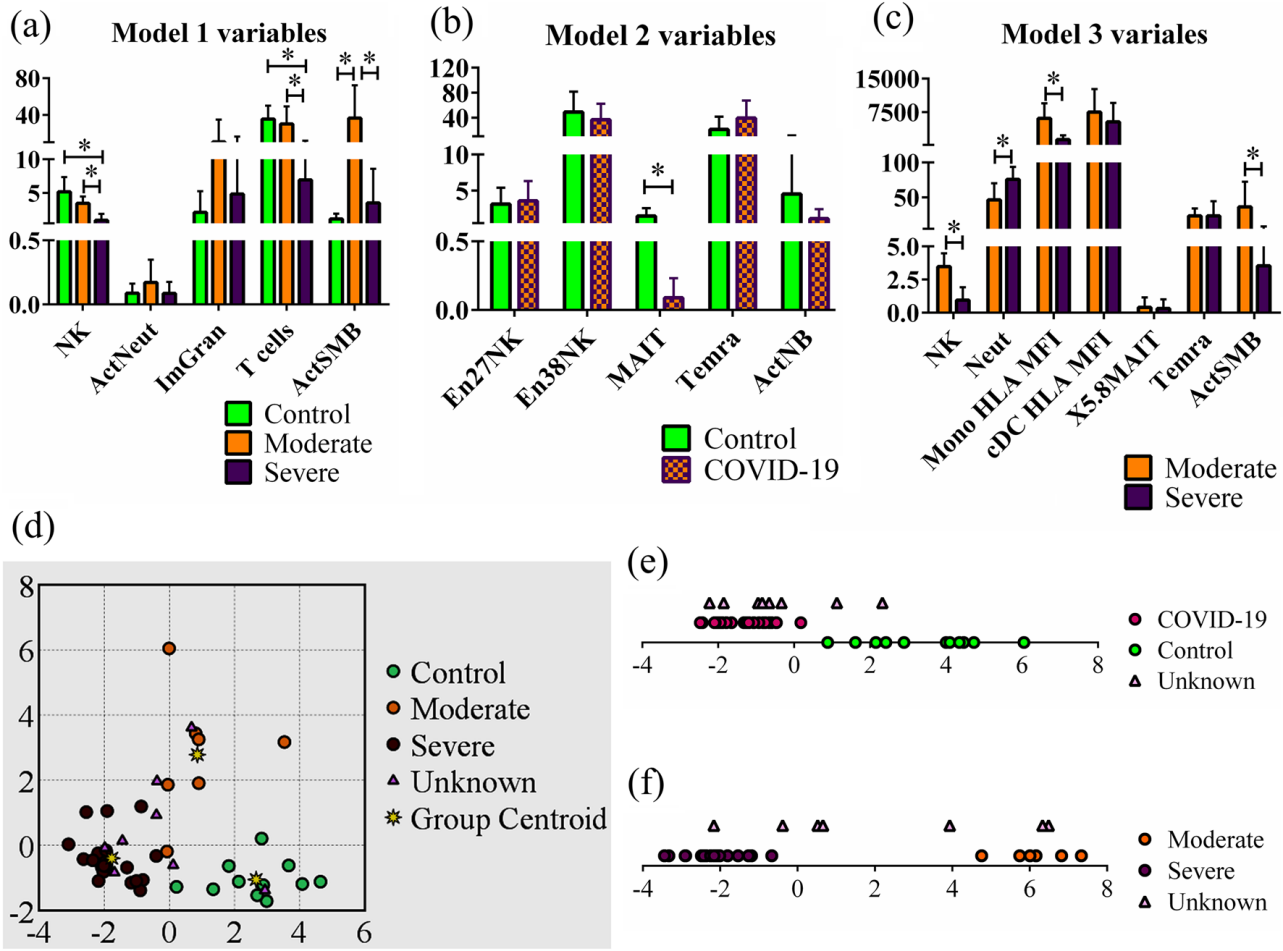
Discriminant analysis-based distinction between COVID-19 patients presenting with different levels of disease severity and healthy volunteers. Discriminant analysis was done using the stepwise method and raw data corresponding to the variables entered in models 1, 2, and 3 are shown in (**a–c**), respectively. (**d**) Model 1: distinction between healthy volunteers (n = 11), moderately ill (n = 7), and severely ill (n = 20) COVID-19 patients. (**e**) Model 2: distinction between healthy volunteers (n = 11) and COVID-19 patients including moderately and severely (n = 27) ill patients combined in one group. (**f**) Model 3: distinction between moderately (n = 7) and severely ill patients (n = 20). Graphs in (**e,f**) are drawn on 1 axis (i.e., the x-axis) and vertical elevation of data points is for illustration purposes only. *NK* natural killer cells, *neut* neutrophils, *actNeut* activated HLA-DR^+^ neutrophils, *imGran* Ki67^+^ immature granulocytes, *Mono HLA MFI* MFI of HLA-DR in monocytes, MFI of HLA-DR in conventional dendritic cells, *MAIT* mucosal associated invariant T cells, *CXCR5*^+^ *CD8*^+^ *MAIT* CXCR5^+^ CD8^+^ mucosal associated invariant T cells, *Temra* CD27^−^CD45RA^+^ effector memory CD8^+^ T cells, *actNB* CD21^+^CD27^−^Ki67^+^ B cells, *actSMB* CD21^−^cd27^+^CD38^lo^ B cells.

The wide range of presentations that develop following infection with SARS-CoV-2 called for a prognostic algorithm that may enable identifying critical patients early after infection. We therefore evaluated three discriminant models of immune profiles to distinguish healthy controls versus moderate or severe disease presentation. One model was designed to distinguish between the three groups of participants: control, moderate, and severe (Model 1). The predictive model was significant (*p* = 4.87 × 10^−15^) with a Wilks’ λ of 0.065, indicating that a majority of the variance contained in the model’s discriminant functions could be explained by differences in group membership. The model was built in five steps, each of which was statistically significant (*p* = 6.68 × 10^−15^–1.11 × 10^−8^) and contributed to improving the model as indicated by the incremental decrease of Wilks’ λ from 0.351 in the first step to 0.065 with the fifth (Table 1). Such small Wilks’ λ is consistent with a good fit with 93.5% of model variance (1 − 0.065 = 0.935) geared toward predicting group membership. The model contained two canonical discriminant functions, the first of which was a more important predictor of group membership than the second, as indicated by the first’s higher eigenvalue (4.155 versus 1.985), larger proportion of variance it explained (67.7% versus 32.3%), and greater canonical correlation (0.898 versus 0.815) (Table 1). Five variables were sequentially incorporated into the model in this order: NK cells, T cells, CD21^−^CD27^+^CD38^lo^ (class-switched, activated memory^27,28^) B cells (actSMB), activated HLA-DR^+^ neutrophils^29^ (actNeut), and Ki67^+^ immature granulocytes (imGran). As seen in the model’s discriminant score plot, the severe and moderate groups were well separated from controls on the first discriminant function, while the moderate group was separated from severe and controls on the second function (Fig. 1d). The first discriminant function was most representative of NK cells and T cells, while the second represented actSMB and actNeut the most. ImGran almost equally contributed to both discriminant functions where their contributions ranked third for both functions. Despite model improvements with the introduction of each of the five variables, only the first three variables showed significantly different group means (by ANOVA with Holm correction and pairwise comparisons using a t-test with Holm-Sidak correction) and had relatively small Wilks’ λs (Table 2), prompting us to conclude that some of the variables ruled nonpromising based on individual biomarker evaluations, may still be useful to the model. Pairwise comparisons using a t-test p-value cutoff of 0.05 showed that NK cells and T cells were less abundant in patients with severe COVID-19 compared to uninfected controls and moderate disease patients, while ActSMB were more abundant in patients with moderate disease compared to those with severe disease and uninfected controls (Fig. 1a). In conclusion, model 1 proposes that reduced frequency of NK cells and T cells are the most distinguishing features separating severe COVID-19 from moderate COVID-19 and controls, high frequency of actSMB and actNeut are the most distinguishing features separating moderate COVID-19 from severe COVID-19 and controls, and imGran play a minor role in separating the three groups.

**Table 2:** Optimized multivariate immune profiles for predicting COVID-19 disease severity using discriminant analysis.

	Wilks’ λ	*p-*value*/*corrected* p-*value	Standardized canonical discriminant function coefficient	
			1st discriminant function	2nd discriminant function
Model 1				
NK cells	0.351	1.11 × 10^−8^/1.89 × 10^−6^	0.886	0.019
ActSMB	0.559	3.84 × 10^−5^/6.21 × 10^−3^	− 0.049	1.242
T cells	0.418	2.33 × 10^−7^/3.96 × 10^−5^	0.706	− 0.176
ActNeut	0.907	0.181/1	− 0.042	0.825
ImGran	0.953	0.432/1	− 0.451	0.437
Model 2				
MAIT	0.459	1.434 × 10^−7^/2.44 × 10^−5^	1.024	N/A
En38NK	0.960	2.31 × 10^−3^/0.365	0.968	N/A
ActNB	0.852	0.017/1	0.984	N/A
En27NK	0.993	0.629/1	− 0.626	N/A
Temra	0.908	0.063/1	− 0.443	N/A
Model 3				
Monocyte HLA-DR MFI	0.413	3.17 × 10^−6^/5.42 × 10^−4^	0.616	N/A
ActSMB	0.597	3.72 × 10^−4^/0.0617	1.415	N/A
NK cells	0.420	3.90 × 10^−6^/6.63 × 10^−4^	1.199	N/A
CXCR5^+^ CD8^+^ MAIT	0.997	0.786/1	1.493	N/A
cDC HLA-DR MFI	0.952	0.271/1	0.944	N/A
Temra	1.000	0.998/1	− 1.137	N/A
Neutrophils	0.681	2.14 × 10^−3^/0.352	− 0.622	N/A

Discriminant models were constructed to distinguish between healthy volunteers, moderately ill patients, and severely ill patients (Model 1); healthy volunteers and COVID-19 patients presenting with moderate or severe disease (Model 2); or moderately ill and severely ill COVID-19 patients (Model 3). Model variables are listed in the order they were incorporated into the model. Correction for multiple testing was performed using Holm method^60^.

*NK cells* natural killer cells, *en27NK* CD27^+^ NK cells, *en38NK* CD38^+^ NK cells, *actNeut* activated HLA-DR^+^ neutrophils, *imGran* Ki67^+^ immature granulocytes, *Temra* CD27^−^CD45RA^+^ effector memory CD8^+^ T cells, *actNB* CD21^+^CD27^−^Ki67^+^ B cells, *actSMB* CD21^−^CD27^+^CD38^lo^ B cells.

The second model (Model 2) was designed to distinguish between healthy volunteers and COVID-19 patients, regardless of the latter’s disease severity status. The model was statistically significant (*p* = 1.10 × 10^−11^) and had a good fit as a predictive model with a Wilks’ λ of 0.166. Since this model aimed to distinguish between two groups, only one discriminant function was extracted, which explained 100% of variance and had an eigenvalue of 5.03 and a canonical correlation of 0.913 (Table 1). This model was also compiled in five steps, each of which was significant (*p* = 1.36 × 10^−11^–1.43 × 10^−7^) and improved the model as indicated by the incremental decline of Wilks’ λ starting at 0.459 in the first step and reaching 0.166 in the last (Table 1). Five variables were included in the model, of which MAIT (decreased in COVID-19 patients compared to control) was the only significantly different variable (Fig. 1b). More than 50% of the variance of this variable could be explained by group membership, as indicated by a Wilks’ λ of 0.459. Having the highest standardized canonical discriminant function coefficient value (1.024), MAIT population was the most impactful on the single discriminant function in Model 2 and, thus, on group separation. Group means did not significantly differ for the remaining four variables [CD38^+^ NK cells (NK cells with enhanced cytotoxicity and cytokine secretion^30^) (en38NK), CD21^+^CD27^−^Ki67^+^ B cells (proliferating/activated naïve B cells^31,32^) (actNB), CD27^+^ NK cells (NK cells with enhanced function^33,34^) (en27NK), and CD27^−^CD45RA^+^ effector memory CD8^+^ T cells (terminally differentiated effector T cells^35^) (Temra)]; these variables had relatively high Wilks’ λs (0.960, 0.852, 0.993, and 0.908, respectively), indicating that only small portions of their respective variances were related to group membership (Table 2). However, these variables were not useless to the model since incorporating each of them resulted in a highly significant boost to the discriminatory ability of the model—as indicated by the *p*-values associated with each step (see above and Table 1)—and a decrease in the model’s Wilks’ λ (Table 1). En38NK and actNB (standardized canonical discriminant function coefficients of 0.968 and 0.684, respectively) impacted the sole discriminant function of the model more than en27NK and Temra did (standardized canonical discriminant function coefficients of − 0.626 and − 0.443, respectively) (Table 2). In brief, the model suggests that the lower frequency of MAIT is the most prominent distinguishing feature that separates COVID-19 patients from controls, while en27NK, en38NK, actNB, and Temra improve the discriminant model despite a lack of significant differences between groups.

The last model (Model 3) aimed to distinguish COVID-19 patients with severe disease from those with moderate presentation. The model was statistically significant (8.73 × 10^−11^) with an excellent fit (Wilks’ λ of 0.058). The model was constructed in seven steps, all of which were highly significant (*p* = 2.01 × 10^−11^–3.00 × 10^−6^) and resulted in a corresponding decrease in Wilks’ λ. The model’s single discriminant function explained 100% of variance and had an eigenvalue of 16.219 and canonical correlation of 0.971 (Table 1). Multiple aspects of this model were paradoxical. Group means were significantly different for two variables (NK cells and the mean fluorescence intensity (MFI) of HLA-DR on monocytes) when correcting for multiple testing over all variables, and four variables (additional two variables were neutrophils and actSMB) when correcting for multiple testing over the number of variables incorporated in the model (Fig. 1c). However, these variables were not the most impactful on the discriminant function of the model. In fact, monocytes’ HLA-DR MFI and neutrophils frequency had the lowest absolute standardized canonical discriminant function coefficient (0.616) and, thus, the smallest impact compared to other variables in the model. NK cells and actSMB had a standardized canonical discriminant function coefficient of 1.199 and 1.415, making them the second and fourth most impactful in the model, respectively. On the other hand, CXCR5^+^ CD8^+^ MAIT and Temra had the strongest and third strongest impacts on the discriminant function (standardized canonical discriminant function coefficients of 1.493 and − 1.137), respectively, but none of them had significantly different group means. Even more perplexing is that almost none of CXCR5^+^ CD8^+^ MAIT and Temra variance was related to group membership (Wilks’ λ of 0.997 and 1.000, respectively) (Table 2). It is noteworthy that MFI of HLA-DR on monocytes had the smallest Wilks’ λ (0.413) of any variable tested in the study, thus, using it as a nidus around which the model was built was, indeed, appropriate. The fact that six other variables were added in the following steps indicates that each introduced variable lowered the models’ Wilks’ λ the most at the step in which it was introduced, which is mandated by the algorithm. In conclusion, it appears that decreased frequency of NK cells and actSMB, decreased MFI of monocyte HLA-DR, and increased frequency of neutrophils are the main distinguishing features of severe COVID-19 that set it apart from COVID-19 of moderate severity.

To visually observe group separation using the three models, we examined the corresponding canonical score plots. Model 1 clearly separated uninfected controls from moderate and severe patients, while the latter two appeared closer to each other than either of them was to the control. This finding suggested that distinguishing between moderate and severe patients would probably be more challenging than distinguishing between infected and uninfected participants, which will be addressed below (Fig. 1d). Model 2 plot shows complete separation between COVID-19 patients—inclusive of patients with moderate and severe disease—and uninfected controls. Severe and moderate patients overlapped, almost completely, as expected since they were all in one group whose centroid and the centroid of the control group defined the direction of the models’ discriminant function (Fig. 1e). Model 3 shows effective separation between moderate and severe patients (Fig. 1f).

### Creating binary logistic regression models for binary dependent variables

Models 2 and 3 were recreated using binary logistic regression resulting in two new models that we named models 2′ and 3′, respectively. According to Chi-square test results, both new models were highly significant (*p* = 1.18 × 10^−10^ and *p* = 2.26 × 10^−7^, respectively). The Hosmer–Lemeshow null hypothesis of perfect group-membership prediction was retained at *p* = 1.000 for each of the two steps in model 2′ and the one step of Model 3′. All steps in both models had very high Nagelkerke’s pseudo-*R*^2^ values (0.916–1.000) (Table 3). These data strongly suggest that each of the two models had a strong predictive power. In Model 2′, there were 27 COVID-19 patients and 11 controls with the COVID-19 group being the target group—meaning we were interested in estimating the odds and probability of having COVID-19 for each of our participants. MAIT was introduced to the model in the first step and the corresponding Chi-square *p*-value was 4.45 × 10^−10^ (Table 3), implying that the model at this stage was highly likely to be a better predictor of group membership than the null model containing no predictor variables. In the second step, which was also significant (*p* = 0.009), the CD56^dim^CD16^+^ NK cells variable was introduced (Table 3). No more steps or variables were added to the model signaling that it could not be significantly improved by incorporating more variables. Model 3′ had seven moderately ill and twenty severely ill patients, with the severely ill being the target group. Only one predictor variable—MFI of HLA-DR on monocytes—was introduced into the model with a Chi-square *p*-value of 2.26 × 10^−7^. Regression weights, significance of each predictor variable, and odds ratios were not reliably calculated due to complete or quasi-complete group separation. This issue will be addressed in the "Discussion" section. The differences in the makeup of the logistic regression models compared to the corresponding discriminate models led us to put each of these models to the test and empirically determine their predictive power.

**Table 3:** Optimized multivariate immune profiles for predicting COVID-19 disease severity using binary logistic regression.

	Chi-square* p*-value	HL test	*R*^2^	Variables entered (regression weights/*p-*value)	Odds ratio
Model 2′					
Step 1	4.4486 × 10^−10^	1.000	0.916	MAIT (12.859/0.060)	3.843 × 10^5^
Step 2	9.00 × 10^−3^	1.000	1.000	CD56^dim^CD16^+^ NK cells (0.964/0.994) MAIT (102.525/0.993)	2.623 3.358 × 10^44^
Model	1.1759 × 10^−10^	N/A	N/A	N/A	N/A
Model 3′					
Step 1	2.2555 × 10^−7^	1.000	0.923	Monocyte HLA-DR MFI (0.007/0.225)	1.007
Model	2.2555 × 10^−7^	N/A	N/A	N/A	N/A

*R*^*2*^ Nagelkerke’s pseudo-*R*^*2*^, *HL* Hosmer–Lemeshow test.

### Evaluation of the discriminant and binary logistic regression models

**Figure 2:**
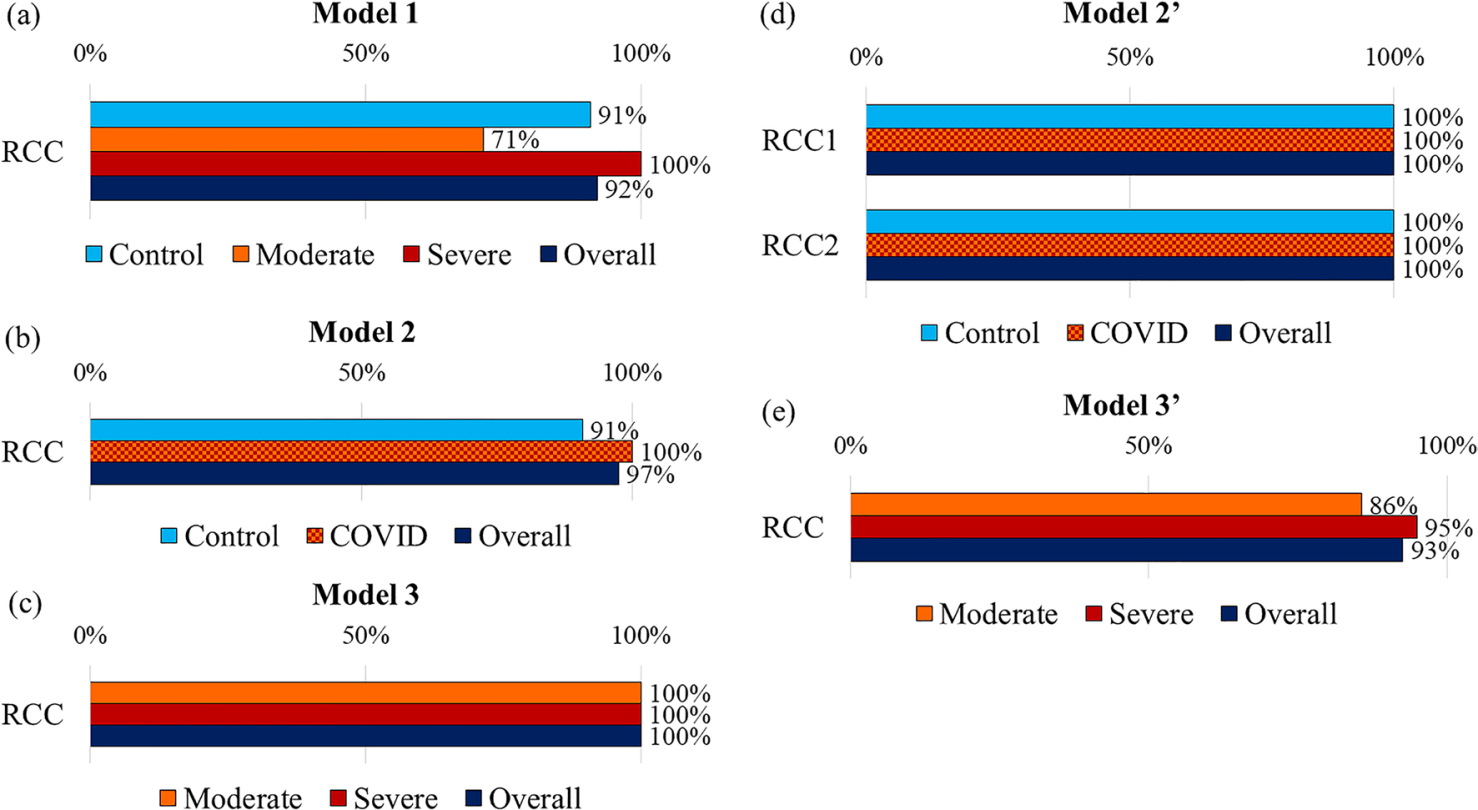
Rate of correct classification (RCC) based on the discriminant models (models 1, 2, and 3) and binary logistic regression models (models 2′ and 3′). (**a**) RCC for Model 1, distinguishing between healthy volunteers (n = 11), the moderately ill (n = 7), and the severely ill (n = 20) COVID-19 patients. (**b**) RCC for Model 2, distinguishing between healthy volunteers (n = 11) and COVID-19 patients including moderately and severely ill patients combined in one group (n = 27). (**c**) RCC for Model 3, distinguishing between moderately (n = 7) and severely ill (n = 20) patients. (**d**) RCC for Model 2′, distinguishing between healthy volunteers (n = 11) and COVID-19 patients (n = 27). (**e**) RCC for Model 3′, distinguishing between moderately (n = 7) and severely ill (n = 20) patients. RCC1 and RCC2 in Model 2′ are the RCCs for steps 1 and 2 of the logistic regression models, respectively.

RCC was used to evaluate each models’ ability to correctly assign participants to their respective groups. Our discriminant models were more successful in correctly classifying participants into two groups than three groups. Model 1 achieved 92% overall RCC with RCCs of 91%, 71%, and 100% for healthy controls, the moderately ill, and the severely ill groups, respectively. Model 2 achieved an overall RCC of 97%, 91% for the control group, and 100% for the COVID-19 group. Model 3 achieved 100% RCC for both moderate and severe groups. Model 2′ showed 100% RCC for both the control and COVID-19 groups, which was achieved even with one variable (MAIT) in the model. Model 3′ showed an overall RCC of 93%, 86% for the moderate group, and 95% for the severe groups (Fig. 2).

**Figure 3:**
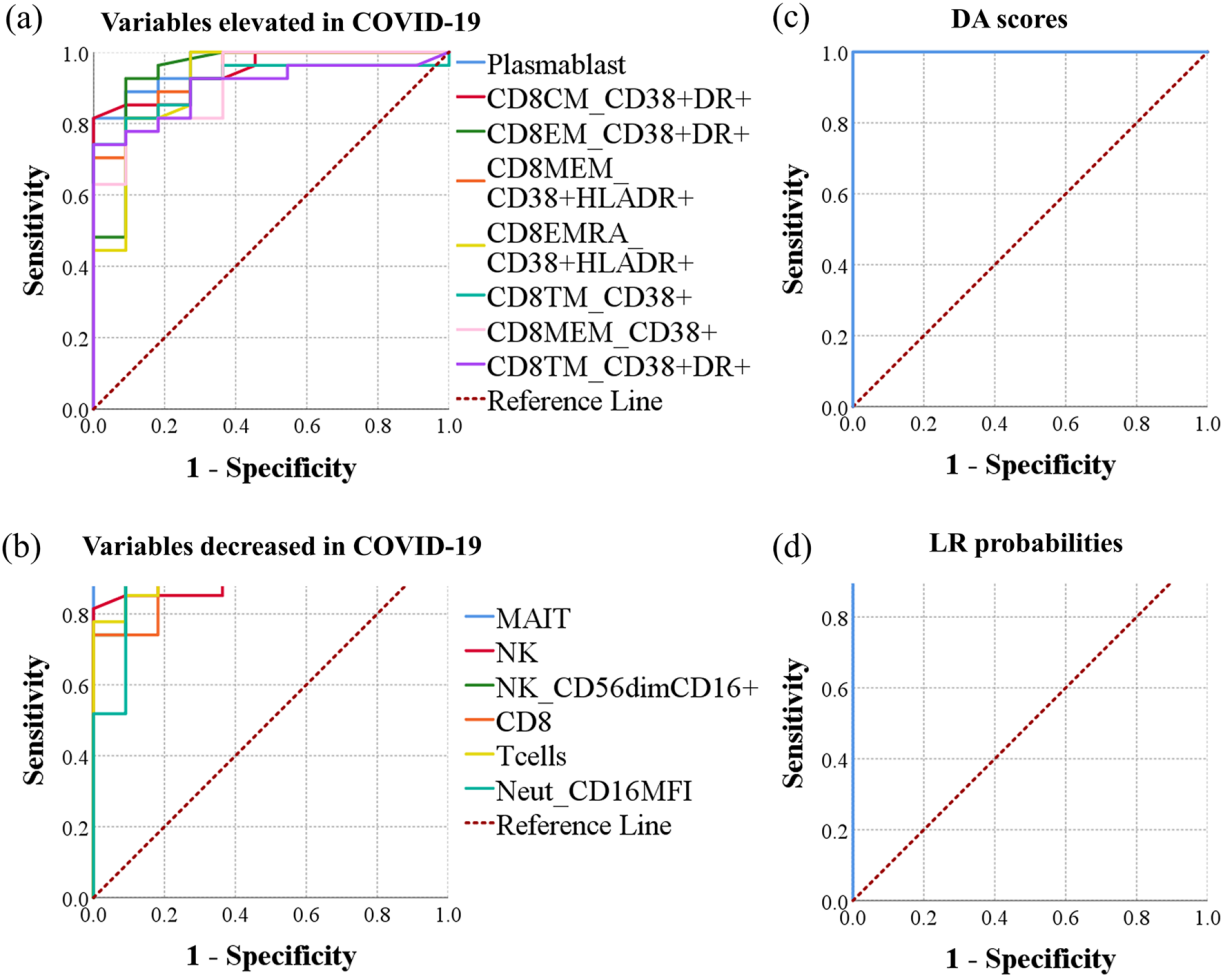
Evaluation of individual predictors, discriminant scores, and binary logistic regression probabilities as biomarkers of COVID-19 among a group of patients and normal controls. ROC curves were generated using data from healthy control participants (n = 12) and patients with moderate or severe disease (n = 34). ROC curves of (**a**) individual analytes elevated in COVID-19, (**b**) individual analytes decreased in COVID-19, (**c**) discriminant function scores, and (**d**) probabilities of having COVID-19 computed using binary logistic regression are shown.

We also used the AUC method to compare the predictive ability of the two-group models (Models 2, 3, 2′, and 3′) to each other and to the use of individual predictor variables. In distinguishing between healthy participants and COVID-19 patients regardless of disease severity status, the largest AUC of any individual variable was that of the frequency of MAIT cells (0.993). Plasmablasts, three populations of CD38^+^ HLA-DR^+^ CD8^+^ T cells (central memory, effector memory, and total memory), and NK cells had the second through sixth largest AUCs (0.966, 0.946, 0.943, 0.939, and 0.931, respectively) among all individual analytes. Combining biomarkers using DA scores or binary logistic regression probabilities resulted in maximum AUCs of 1.000, indicating an improved predictive power with the use of multivariate biomarkers (Fig. 3, Table 4).

**Table 4:** Estimating the predictive ability of various analytes in identifying COVID-19 patients, regardless of disease severity status using the area under a receiver operating characteristic curve (AUC) method.

Variable	Area	*p-*value	Asymptotic 95% confidence interval	
			Lower bound	Upper bound
**Combined profiles**				
Discriminant scores	1.000	2 × 10^−6^	1.000	1.000
BLR probabilities	1.000	2 × 10^−6^	1.000	1.000
**AUC (analytes elevated in COVID-19)**				
Plasmablast	0.966	8.28 × 10^−6^	0.917	1.000
CD38^+^ HLA-DR^+^ CD8^+^ central memory T cells	0.946	2.00 × 10^−5^	0.881	1.000
CD38^+^ HLA-DR^+^ CD8^+^ effector memory	0.943	2.31 × 10^−5^	0.850	1.000
CD38^+^ HLA-DR^+^ CD8^+^ memory	0.939	2.66 × 10^−5^	0.866	1.000
CD38^+^ HLA-DR^+^ CD8^+^ CD45RA^+^ effector memory	0.918	6.57 × 10^−5^	0.812	1.000
CD38^+^ CD8^+^ transitional memory	0.916	7.04 × 10^−5^	0.825	1.000
CD38^+^ CD8^+^ memory	0.912	8.05 × 10^−5^	0.819	1.000
CD38^+^ HLA-DR^+^ CD8^+^ transitional memory	0.904	1.12 × 10^−4^	0.808	1.000
CD38^+^ HLA-DR^+^ circulating T-follicular helper	0.894	1.66 × 10^−4^	0.796	0.992
CD38^+^ HLA-DR^+^ CD4^+^ memory	0.889	2.01 × 10^−4^	0.786	0.992
CD38^+^ CD8^+^ effector memory	0.887	2.14 × 10^−4^	0.780	0.995
CD38^+^ HLA-DR^+^ CD4^+^ central memory	0.879	2.93 × 10^−4^	0.772	0.985
CD27^+^ CD38^+^ HLA-DR^+^ CD8^+^ CD45RA^+^ effector memory	0.872	3.76 × 10^−4^	0.751	0.993
CD38^+^ CD4 + transitional memory	0.855	6.84 × 10^−4^	0.700	1.000
CD38^+^ HLA-DR^+^ CD4^+^ effector memory	0.855	6.84 × 10^−4^	0.722	0.988
CD38^+^ HLA-DR^+^ CD4^+^ transitional memory	0.854	7.26 × 10^−4^	0.732	0.975
CD38^+^ CD8^+^ CD45RA^+^ effector memory	0.845	9.70 × 10^−4^	0.717	0.973
CD21^−^ CD27^−^ CD38^lo^ B cells	0.838	1.22 × 10^−3^	0.696	0.981
CD38^+^ CD8^+^ MAIT	0.830	1.61 × 10^−3^	0.701	0.959
CD27^−^ CD38^+^ HLA-DR^+^ CD8^+^ CD45RA^+^ effector memory	0.825	1.90 × 10^−3^	0.692	0.957
CD38^+^ CD4^+^ effector memory	0.825	1.90 × 10^−3^	0.677	0.973
Neutrophils	0.825	1.90 × 10^−3^	0.692	0.958
CD27^+^ CD38^+^ CD8^+^ CD45RA^+^ effector memory	0.822	2.11 × 10^−3^	0.684	0.959
Eosinophils	0.813	2.76 × 10^−3^	0.679	0.947
CD38^+^ CD8^+^ central memory	0.808	3.23 × 10^−3^	0.638	0.978
PD1^+^ CD8^+^ transitional memory	0.808	3.23 × 10^−3^	0.673	0.943
HLA-DR^+^ CD8^+^ memory	0.801	3.97 × 10^−3^	0.662	0.940
HLA-DR^+^ circulating T-follicular helper	0.801	3.97 × 10^−3^	0.660	0.943
CD38^+^ HLA-DR^+^ CD8^+^ MAIT	0.800	4.17 × 10^−3^	0.660	0.939
HLA-DR^+^ CD8^+^ effector memory	0.795	4.86 × 10^−3^	0.655	0.934
CD38^+^ CD4 + memory	0.793	5.11 × 10^−3^	0.616	0.970
HLA-DR^+^ CD8^+^ MAIT	0.791	5.37 × 10^−3^	0.651	0.931
CD27^−^ CD38^+^ CD8^+^ CD45RA^+^ effector memory	0.774	8.71 × 10^−3^	0.626	0.923
CD27^−^HLA-DR^+^ CD8^+^ CD45RA^+^ effector memory	0.771	9.57 × 10^−3^	0.624	0.918
CD27^−^ HLA-DR^+^ CD8^+^ CD45RA^+^ effector memory	0.768	1.05 × 10^−2^	0.617	0.918
PD1^+^ CD4^+^ effector memory	0.758	1.38 × 10^−2^	0.606	0.909
CD25^+^ CD8^+^ memory	0.756	1.44 × 10^−2^	0.600	0.912
HLA-DR^+^ CD8^+^ transitional memory	0.747	1.80 × 10^−2^	0.594	0.901
PD1^+^ CD4^+^ transitional memory	0.747	1.80 × 10^−2^	0.595	0.900
PD1^+^ CD4^+^ memory	0.746	1.88 × 10^−2^	0.591	0.900
CD25^+^ CD8^+^ central memory	0.744	1.96 × 10^−2^	0.582	0.906
HLA-DR^+^ CD8^+^ central memory	0.734	2.53 × 10^−2^	0.570	0.898
CD25^+^ CD8^+^ effector memory	0.734	2.53 × 10^−2^	0.569	0.899
CD25^+^ CD8^+^ transitional memory	0.734	2.53 × 10^−2^	0.566	0.902
CD27^+^ HLA-DR^+^ CD8^+^ CD45RA^+^ effector memory	0.729	2.86 × 10^−2^	0.567	0.891
HLA-DR^+^ CD4^+^ memory	0.714	4.10 × 10^−2^	0.548	0.880
CD38^+^ CD4^+^ central memory	0.704	5.15 × 10^−2^	0.502	0.906
HLA-DR^+^ CD4^+^ central memory	0.704	5.15 × 10^−2^	0.536	0.872
CD27^−^ CD8^+^ T cells	0.700	5.55 × 10^−2^	0.528	0.873
Immature granulocyte	0.699	5.76 × 10^−2^	0.532	0.865
CD69^+^ CD8^+^ MAIT	0.697	5.97 × 10^−2^	0.528	0.865
PD1^+^ CD8^+^ MAIT	0.690	6.90 × 10^−2^	0.504	0.876
PD1^+^ CD4 + central memory	0.684	7.94 × 10^−2^	0.514	0.853
CD21^−^ CD27^+^ CD38^lo^ B cells	0.675	9.42 × 10^−2^	0.494	0.856
HLA-DR^+^ CD4 + effector memory	0.668	1.08 × 10^−1^	0.478	0.859
CD27^−^ CD8^+^ CD45RA effector memory	0.667	1.11 × 10^−1^	0.495	0.839
Monocytes	0.657	1.34 × 10^−1^	0.427	0.886
CD25^+^ CD27^+^ CD8^+^ CD45RA^+^ effector memory	0.646	1.61 × 10^−1^	0.453	0.840
Classical monocytes	0.645	1.66 × 10^−1^	0.426	0.864
Intermediate monocytes	0.645	1.66 × 10^−1^	0.439	0.850
CD8^+^ CD45RA^+^ effector memory	0.643	1.71 × 10^−1^	0.453	0.833
PD1^+^ CD8^+^ central memory	0.641	1.76 × 10^−1^	0.451	0.832
PD1^+^ CD8^+^ memory	0.636	1.92 × 10^−1^	0.455	0.818
Ki67^+^ CD8^+^ memory	0.633	2.04 × 10^−1^	0.446	0.820
CXCR5^+^ CD4^+^ transitional memory	0.626	2.27 × 10^−1^	0.429	0.824
HLA-DR^+^ CD4^+^ transitional memory	0.626	2.27 × 10^−1^	0.442	0.810
CD25^+^ NK cells	0.625	2.34 × 10^−1^	0.438	0.811
Plasmacytoid dendritic cells DC HLA-DR MFI	0.618	2.60 × 10^−1^	0.386	0.850
Ki67^+^ CD8^+^ central memory	0.616	2.67 × 10^−1^	0.434	0.798
Intermediate monocytes/monocytes	0.606	3.11 × 10^−1^	0.412	0.800
Ki67^+^ CD8^+^ effector memory	0.604	3.18 × 10^−1^	0.417	0.791
CD4^+^ central memory	0.593	3.76 × 10^−1^	0.403	0.782
CXCR5^+^ CD8^+^ effector memory	0.588	4.03 × 10^−1^	0.409	0.766
CD69^+^ CD4^+^ memory	0.584	4.21 × 10^−1^	0.390	0.778
CD25^+^CD27^−^ CD8^+^ CD45RA^+^ effector memory	0.572	4.89 × 10^−1^	0.383	0.762
CD4^+^ Tregs	0.569	5.09 × 10^−1^	0.390	0.748
CD27^+^ CD8^+^ T cells	0.559	5.73 × 10^−1^	0.369	0.749
CD69^+^ CD4^+^ effector memory	0.556	5.95 × 10^−1^	0.369	0.743
Ki67^+^ CD4^+^ memory	0.551	6.29 × 10^−1^	0.364	0.737
Ki67^+^ CD21^−^ CD27^−^ B cells	0.547	6.52 × 10^−1^	0.325	0.769
CD27^−^CXCR5^+^ CD8^+^ CD45RA^+^ effector memory	0.542	6.87 × 10^−1^	0.340	0.744
PD1^+^ CD8^+^ effector memory	0.542	6.87 × 10^−1^	0.342	0.742
CD4^+^ transitional memory	0.539	7.11 × 10^−1^	0.339	0.739
Non-classical monocytes	0.539	7.11 × 10^−1^	0.310	0.768
CXCR5^+^ CD4^+^ effector memory	0.537	7.23 × 10^−1^	0.340	0.734
CD25^+^ CD8^+^ CD45RA^+^ effector memory	0.537	7.23 × 10^−1^	0.341	0.733
PD1^+^ CD8^+^ CD45RA^+^ effector memory	0.535	7.35 × 10^−1^	0.340	0.730
Ki67^+^ CD4^+^ effector memory	0.525	8.09 × 10^−1^	0.330	0.720
CD4^+^ CD45RA^+^ effector memory	0.525	8.09 × 10^−1^	0.333	0.717
Circulating T-follicular helper	0.525	8.09 × 10^−1^	0.328	0.722
CD27^+^ NK cells	0.522	8.34 × 10^−1^	0.331	0.712
Ki67^+^ CD8^+^ transitional memory	0.520	8.47 × 10^−1^	0.335	0.705
CD69^+^ CD4^+^ transitional memory	0.515	8.85 × 10^−1^	0.324	0.706
CD27^+^ Ki67^+^ CD8^+^ CD45RA^+^ effector memory	0.515	8.85 × 10^−1^	0.323	0.707
CD25^+^ CD8^+^ MAIT	0.515	8.85 × 10^−1^	0.334	0.697
CD27^+^CD69^+^ CD8^+^ CD45RA^+^ effector memory	0.508	9.36 × 10^−1^	0.330	0.686
CD27^+^ CD8^+^ CD45RA^+^ effector memory	0.508	9.36 × 10^−1^	0.305	0.712
Ki67^+^ CD8^+^ MAIT	0.505	9.61 × 10^−1^	0.325	0.685
CXCR5^+^ CD8^+^ transitional memory	0.503	9.74 × 10^−1^	0.309	0.697
CD27^−^Ki67^+^ CD8^+^ CD45RA^+^ effector memory	0.503	9.74 × 10^−1^	0.309	0.698
Eosinophils CD15 MFI	0.502	9.87 × 10^−1^	0.296	0.707
CD4^+^ effector memory	0.502	9.87 × 10^−1^	0.307	0.697
Classical monocytes/monocytes	0.502	9.87 × 10^−1^	0.283	0.720
CD8^+^ central memory	0.500	1.00	0.290	0.710
**AUC (analytes decreased in COVID-19)**				
MAIT	0.993	2.41 × 10^−6^	0.976	1.000
NK cells	0.931	3.79 × 10^−5^	0.854	1.000
CD56^dim^ CD16^+^ NK cells	0.926	4.67 × 10^−5^	0.839	1.000
CD8^+^ T cells	0.909	9.20 × 10^−5^	0.818	1.000
Neutrophil CD16 MFI	0.902	1.20 × 10^−4^	0.797	1.000
T cells	0.902	1.20 × 10^−4^	0.803	1.000
Innate lymphoid cells	0.879	2.93 × 10^−4^	0.771	0.987
NK cells CD16 MFI	0.845	9.70 × 10^−4^	0.718	0.972
CD4^+^ T cells	0.842	1.09 × 10^−3^	0.714	0.969
B cells	0.842	1.09 × 10^−3^	0.709	0.974
CD21^+^ CD27^+^ CD38^lo^ B cells	0.842	1.09 × 10^−3^	0.717	0.966
CD56^hi^ CD16^−^ NK cells	0.816	2.48 × 10^−3^	0.682	0.951
CD38^+^ NK cells	0.778	7.92 × 10^−3^	0.617	0.939
CD8^+^ transitional memory	0.729	2.86 × 10^−2^	0.561	0.897
Neutrophils CD15 MFI	0.727	2.98 × 10^−2^	0.555	0.899
CD21^+^ CD27^−^ CD38^lo^ B cells	0.721	3.50 × 10^−2^	0.552	0.889
CD69^+^ CD8 + transitional memory	0.717	3.79 × 10^−2^	0.556	0.879
Plasmacytoid dendritic cells	0.712	4.26 × 10^−2^	0.528	0.896
Conventional dendritic cells	0.710	4.43 × 10^−2^	0.518	0.903
PD1^+^ NK cells	0.704	5.15 × 10^−2^	0.533	0.874
Ki67^+^ NK cells	0.684	7.94 × 10^−2^	0.484	0.883
Monocyte CD16 MFI	0.684	7.94 × 10^−2^	0.476	0.891
Monocyte CD14 MFI	0.684	7.94 × 10^−2^	0.476	0.891
Monocyte HLA-DR MFI	0.673	9.74 × 10^−2^	0.446	0.901
CD16^+^ NK cells	0.665	1.15 × 10^−1^	0.486	0.844
CD21^−^ CD27^+^ Ki67^+^ B cells	0.662	1.22 × 10^−1^	0.463	0.860
CD69^+^ CD8^+^ central memory	0.660	1.26 × 10^−1^	0.488	0.832
Non-classical monocytes/monocytes	0.658	1.30 × 10^−1^	0.479	0.837
CD38^+^ HLA-DR^+^ NK cells	0.652	1.48 × 10^−1^	0.436	0.867
CXCR5^+^ CD8^+^ central memory	0.646	1.61 × 10^−1^	0.452	0.841
Naïve CD4^+^ T cells	0.618	2.60 × 10^−1^	0.423	0.813
CD69^+^ CD8^+^ effector memory	0.616	2.67 × 10^−1^	0.437	0.795
Dendritic cells	0.614	2.74 × 10^−1^	0.413	0.816
CD21^+^ CD27^+^ Ki67^+^ B cells	0.606	3.11 × 10^−1^	0.400	0.812
CD16^+^ monocytes	0.603	3.26 × 10^−1^	0.387	0.818
CD38^+^ NK cells	0.599	3.42 × 10^−1^	0.361	0.838
CD16^+^ immature granulocyte	0.599	3.42 × 10^−1^	0.416	0.783
HLA-DR^+^ NK cells	0.599	3.42 × 10^−1^	0.398	0.801
Dendritic cell HLA-DR MFI	0.596	3.59 × 10^−1^	0.339	0.853
CD21^+^ CD27^−^ Ki67^+^ B cells	0.586	4.12 × 10^−1^	0.365	0.806
CD11c^+^ immature granulocyte	0.586	4.12 × 10^−1^	0.394	0.778
Naïve CD8^+^ T cells	0.582	4.30 × 10^−1^	0.387	0.778
CD69^+^ CD8^+^ memory	0.579	4.49 × 10^−1^	0.401	0.757
CXCR5^+^ CD8^+^ MAIT	0.579	4.49 × 10^−1^	0.400	0.758
Ki67^+^ immature granulocyte	0.574	4.79 × 10^−1^	0.392	0.757
CD8^+^ effector memory	0.572	4.89 × 10^−1^	0.374	0.771
CXCR5^+^ CD4^+^ central memory	0.571	4.99 × 10^−1^	0.369	0.773
CXCR5^+^ CD8^+^ CD45RA^+^ effector memory	0.567	5.20 × 10^−1^	0.358	0.777
Conventional dendritic cell HLA-DR MFI	0.564	5.41 × 10^−1^	0.327	0.801
CD123^+^ immature granulocyte	0.559	5.73 × 10^−1^	0.371	0.747
CXCR5^+^ CD4^+^ memory	0.554	6.07 × 10^−1^	0.358	0.750
CD161^+^ monocytes	0.551	6.29 × 10^−1^	0.329	0.772
CD69^+^ CD8^+^ CD45RA^+^ effector memory	0.544	6.76 × 10^−1^	0.363	0.724
CD27^+^CXCR5^+^ CD8^+^ CD45RA^+^ effector memory	0.542	6.87 × 10^−1^	0.342	0.742
Ki67^+^ CD8^+^ CD45RA^+^ effector memory	0.532	7.60 × 10^−1^	0.345	0.719
CD27^−^ CD69^+^ CD8^+^ CD45RA^+^ effector memory	0.530	7.72 × 10^−1^	0.339	0.722
Ki67^+^ neutrophils	0.529	7.84 × 10^−1^	0.331	0.727
CXCR5^+^ CD8^+^ T cells	0.525	8.09 × 10^−1^	0.323	0.727
CXCR5^+^ CD8^+^ memory	0.525	8.09 × 10^−1^	0.329	0.722
Immature granulocyte CD16 MFI	0.515	8.85 × 10^−1^	0.313	0.717
Ki67^+^ CD4^+^ transitional memory	0.515	8.85 × 10^−1^	0.328	0.702
CD27^−^ PD1^+^ CD8^+^ CD45RA^+^ effector memory	0.512	9.10 × 10^−1^	0.311	0.712
CD38^+^ CD161^+^ NK cells	0.512	9.10 × 10^−1^	0.287	0.737
HLA-DR^+^ neutrophils	0.510	9.23 × 10^−1^	0.324	0.696
CD69^+^ CD4^+^ central memory	0.510	9.23 × 10^−1^	0.321	0.699
Ki67^+^ CD4^+^ central memory	0.510	9.23 × 10^−1^	0.320	0.700
B cells HLA-DR MFI	0.502	9.87 × 10^−1^	0.249	0.755

*BLR* binary logistic regression probabilities.

Shown *p*-values are corrected for multiple testing using Holm method. Cell populations are expressed as percentages of their respective parent populations except for where mean fluorescence intensity (MFI) is indicated.

**Figure 4:**
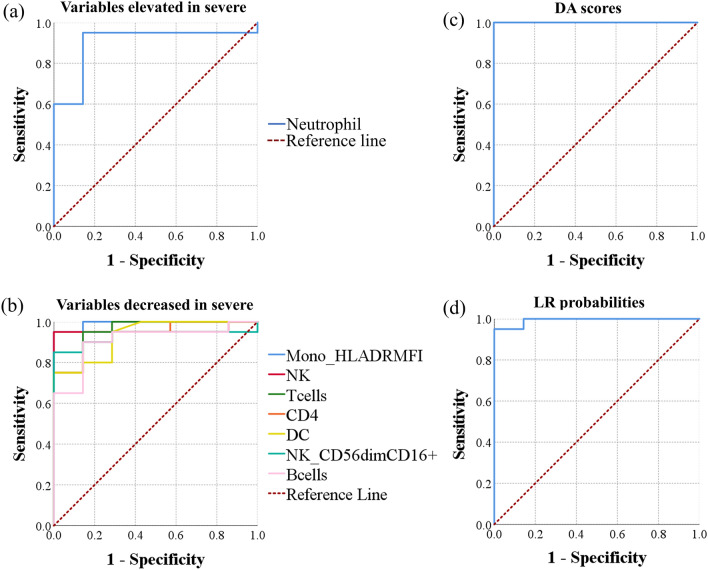
Evaluation of individual biomarkers, discriminant scores, and binary logistic regression probabilities as biomarkers of severe disease among admitted COVID-19 patients. ROC curves were generated using data from patients with moderate disease (n = 7) and patients with severe disease (n = 27). ROC curves of (**a**) individual analytes elevated in severe COVID-19, (**b**) individual analytes decreased in severe COVID-19, (**c**) discriminant function scores, and (**d**) probabilities of having severe disease computed using binary logistic regression are shown.

For predicting severe COVID-19 disease in a pool of hospitalized SARS-CoV-2-positive patients with moderate or severe disease, the largest AUCs of individual biomarkers were obtained using monocyte HLA-DR MFI, frequency of T cells, NK cells, CD4^+^ T cells, dendritic cells, and CD56^dim^ CD16^+^ NK cells with AUCs of 0.993, 0.957, 0.957, 0.936, 0.932, and 0.929, respectively. Combining biomarkers using either DA resulted in a perfect sensitivity and specificity with an AUC of 1.000, while using binary logistic regression was equivalent to monocyte HLA-DR MFI with an AUC of 0.993 (Fig. 4, Table 5).

**Table 5:** Estimating the predictive ability of various analytes in identifying severe COVID-19 patients in a patient population with either moderate or severe disease using the area under a receiver operating characteristic curve (AUC) method.

Variable	Area	*p*-value	Asymptotic 95% confidence interval	
			Lower bound	Upper bound
**Combined profiles**				
Discriminant scores	1.000	1.08 × 10^−4^	1.000	1.000
BLR probabilities	0.993	1.35 × 10^−4^	0.970	1.000
**AUC (analytes elevated in severe COVID-19)**				
Neutrophils	0.900	1.95 × 10^−3^	0.766	1.000
CD27^+^ Ki67^+^ CD8^+^ CD45RA^+^ effector memory	0.871	4.01 × 10^−3^	0.735	1.000
PD1^+^ CD4^+^ transitional memory	0.857	5.67 × 10^−3^	0.717	0.997
CD38^+^ CD4^+^ transitional memory	0.836	9.31 × 10^−3^	0.661	1.000
Ki67^+^ CD8^+^ MAIT	0.836	9.31 × 10^−3^	0.683	0.988
PD1^+^ NK cells	0.829	1.09 × 10^−2^	0.666	0.991
Ki67^+^ CD8^+^ memory	0.821	1.28 × 10^−2^	0.628	1.000
PD1^+^ CD4^+^ memory	0.814	1.49 × 10^−2^	0.652	0.976
PD1^+^ CD8^+^ central memory	0.814	1.49 × 10^−2^	0.649	0.980
CD27^−^ Ki67^+^ CD8^+^ CD45RA^+^ effector memory	0.811	1.61 × 10^−2^	0.646	0.975
Ki67^+^ CD8^+^ CD45RA^+^ effector memory	0.807	1.74 × 10^−2^	0.641	0.973
PD1^+^ CD8^+^ MAIT	0.807	1.74 × 10^−2^	0.613	1.000
Ki67^+^ CD4^+^ central memory	0.796	2.17 × 10^−2^	0.582	1.000
Ki67^+^ CD8^+^ central memory	0.796	2.17 × 10^−2^	0.605	0.988
PD1^+^ CD8^+^ transitional memory	0.793	2.33 × 10^−2^	0.609	0.977
PD1^+^ CD8^+^ memory	0.793	2.33 × 10^−2^	0.603	0.983
CD38^+^ CD4^+^ central memory	0.793	2.33 × 10^−2^	0.619	0.966
PD1^+^ CD8^+^ CD45RA^+^ effector memory	0.779	3.09 × 10^−2^	0.568	0.989
Ki67^+^ CD8^+^ effector memory	0.775	3.32 × 10^−2^	0.595	0.955
PD1^+^ CD4^+^ central memory	0.771	3.55 × 10^−2^	0.592	0.950
Ki67^+^ CD4^+^ memory	0.768	3.80 × 10^−2^	0.551	0.985
CD27^+^ CD38^+^ CD8^+^ CD45RA^+^ effector memory	0.764	4.06 × 10^−2^	0.571	0.957
CD27^−^ PD1^+^ CD8^+^ CD45RA^+^ effector memory	0.757	4.64 × 10^−2^	0.528	0.986
PD1^+^ CD8^+^ effector memory	0.754	4.95 × 10^−2^	0.563	0.944
CD69^+^ CD4^+^ transitional memory	0.746	5.63 × 10^−2^	0.553	0.940
Ki67^+^ NK cells	0.743	5.99 × 10^−2^	0.562	0.924
Ki67^+^ CD8^+^ transitional memory	0.743	5.99 × 10^−2^	0.532	0.954
CD38^+^ CD4^+^ memory	0.736	6.79 × 10^−2^	0.540	0.931
Plasmablasts	0.736	6.79 × 10^−2^	0.486	0.986
CD69^+^ CD4^+^ effector memory	0.725	8.14 × 10^−2^	0.540	0.910
Ki67^+^ CD4^+^ effector memory	0.721	8.63 × 10^−2^	0.514	0.929
CD38^+^ CD8^+^ central memory	0.721	8.63 × 10^−2^	0.512	0.931
CD27^+^ CD69^+^ CD8^+^ CD45RA^+^ effector memory	0.718	9.15 × 10^−2^	0.527	0.909
CD21^+^ CD27^−^ CD38^lo^ B cells	0.714	9.69 × 10^−2^	0.450	0.978
PD1^+^ CD4^+^ effector memory	0.711	1.03 × 10^−1^	0.500	0.921
Classical monocytes/monocytes	0.707	1.09 × 10^−1^	0.517	0.898
CD38^+^ HLA-DR^+^ CD4^+^ central memory	0.704	1.15 × 10^−1^	0.512	0.895
CD38^+^ CD8^+^ transitional memory	0.700	1.21 × 10^−1^	0.476	0.924
HLA-DR^+^ CD4^+^ central memory	0.686	1.50 × 10^−1^	0.491	0.880
Ki67^+^ CD4^+^ transitional memory	0.682	1.58 × 10^−1^	0.463	0.901
CD38^+^ CD4^+^ effector memory	0.664	2.03 × 10^−1^	0.439	0.889
CD38^+^ HLA-DR^+^ CD4^+^ transitional memory	0.664	2.03 × 10^−1^	0.456	0.873
CD4^+^ CD45RA^+^ effector memory	0.661	2.13 × 10^−1^	0.444	0.878
CD38^+^ CD8^+^ memory	0.650	2.45 × 10^−1^	0.425	0.875
Immature granulocyte CD16 MFI	0.650	2.45 × 10^−1^	0.384	0.916
CD27^−^ CD69^+^ CD8^+^ CD45RA^+^ effector memory	0.650	2.45 × 10^−1^	0.447	0.853
CD38^+^ HLA-DR^+^ CD4^+^ memory	0.643	2.68 × 10^−1^	0.442	0.844
CD8^+^ transitional memory	0.639	2.81 × 10^−1^	0.387	0.892
Immature granulocyte CD123^+^	0.636	2.93 × 10^−1^	0.379	0.893
CD4^+^ effector memory	0.636	2.93 × 10^−1^	0.432	0.840
HLA-DR^+^ CD4^+^ memory	0.636	2.93 × 10^−1^	0.429	0.842
HLA-DR^+^ CD4^+^ effector memory	0.636	2.93 × 10^−1^	0.426	0.846
CD69^+^ CD8^+^ CD45RA^+^ effector memory	0.632	3.06 × 10^−1^	0.430	0.835
CD27^+^ CD38^+^ HLA-DR^+^ CD8^+^ CD45RA^+^ effector memory	0.618	3.61 × 10^−1^	0.408	0.828
HLA-DR^+^ NK cells	0.614	3.76 × 10^−1^	0.384	0.844
CD38^+^ HLA-DR^+^ CD4^+^ effector memory	0.614	3.76 × 10^−1^	0.381	0.848
CXCR5^+^ CD8^+^ effector memory	0.607	4.07 × 10^−1^	0.386	0.828
CD69^+^ CD8^+^ MAIT	0.607	4.07 × 10^−1^	0.380	0.834
CD69^+^ CD4^+^ memory	0.604	4.22 × 10^−1^	0.396	0.812
Immature granulocyte CD16^+^	0.600	4.39 × 10^−1^	0.363	0.837
HLA-DR^+^ CD8^+^ central memory	0.600	4.39 × 10^−1^	0.389	0.811
CD27^+^ NK cells	0.600	4.39 × 10^−1^	0.368	0.832
CD38^+^ CD8^+^ effector memory	0.593	4.72 × 10^−1^	0.342	0.844
CD38^+^ CD8^+^ CD45RA^+^ effector memory	0.593	4.72 × 10^−1^	0.324	0.861
HLA-DR^+^ CD4^+^ transitional memory	0.586	5.07 × 10^−1^	0.374	0.797
CD38^+^ HLA-DR^+^ CD8^+^ central memory	0.586	5.07 × 10^−1^	0.374	0.798
CD38^+^ CD8^+^ MAIT	0.579	5.43 × 10^−1^	0.318	0.839
CD38^+^ HLA-DR^+^ T-follicular helper	0.557	6.58 × 10^−1^	0.333	0.781
CD38^+^ HLA-DR^+^ CD8^+^ transitional memory	0.557	6.58 × 10^−1^	0.335	0.780
CD27^+^ HLA-DR^+^ CD8^+^ CD45RA^+^ effector memory	0.557	6.58 × 10^−1^	0.347	0.767
CD27^−^ CD8^+^ CD45RA^+^ effector memory	0.557	6.58 × 10^−1^	0.334	0.780
HLA-DR^+^ T-follicular helper	0.550	6.99 × 10^−1^	0.320	0.780
CD161^+^ monocytes	0.550	6.99 × 10^−1^	0.255	0.845
Ki67^+^ neutrophils	0.543	7.40 × 10^−1^	0.297	0.789
CD27^−^ CD38^+^ CD8^+^ CD45RA^+^ effector memory	0.543	7.40 × 10^−1^	0.270	0.815
Plasmacytoid dendritic cell HLA-DR MFI	0.529	8.25 × 10^−1^	0.291	0.766
HLA-DR^+^ CD8^+^ transitional memory	0.529	8.25 × 10^−1^	0.318	0.739
HLA-DR^+^ CD8^+^ memory	0.525	8.46 × 10^−1^	0.315	0.735
CXCR5^+^ CD4^+^ effector memory	0.521	8.68 × 10^−1^	0.304	0.739
CD69^+^ CD8^+^ memory	0.521	8.68 × 10^−1^	0.310	0.733
CD27^−^ CD8^+^ T cells	0.514	9.12 × 10^−1^	0.302	0.727
CD25^+^ NK cells	0.514	9.12 × 10^−1^	0.255	0.773
Eosinophils CD15 MFI	0.507	9.56 × 10^−1^	0.263	0.751
HLA-DR^+^ CD8^+^ effector memory	0.507	9.56 × 10^−1^	0.286	0.728
CD38^+^ HLA-DR^+^ CD8^+^ effector memory	0.507	9.56 × 10^−1^	0.264	0.751
CD38^+^HLA-DR^+^ NK cells	0.507	9.56 × 10^−1^	0.239	0.775
CD21^+^CD27^+^CD38^lo^ B cells	0.504	9.78 × 10^−1^	0.252	0.756
CD27^+^ CXCR5^+^ CD8^+^ CD45RA^+^ effector memory	0.500	1.00E+00	0.262	0.738
B cells HLA-DR MFI	0.500	1.00E+00	0.256	0.744
**AUC (analytes decreased in severe COVID-19)**				
Monocyte HLA-DR MFI	0.993	1.35 × 10^−4^	0.970	1.000
T cells	0.957	3.99 × 10^−4^	0.879	1.000
NK cells	0.957	3.99 × 10^−4^	0.873	1.000
CD4^+^ T cells	0.936	7.38 × 10^−4^	0.844	1.000
Dendritic cells	0.932	8.16 × 10^−4^	0.835	1.000
CD56^dim^ CD16^+^ NK cells	0.929	9.01 × 10^−4^	0.825	1.000
B cells	0.907	1.61 × 10^−3^	0.786	1.000
CD25^+^ CD8^+^ MAIT	0.879	3.36 × 10^−3^	0.746	1.000
CD25^+^ CD8^+^ transitional memory	0.857	5.67 × 10^−3^	0.706	1.000
CD25^+^ CD8^+^ CD45RA^+^ effector memory	0.843	7.91 × 10^−3^	0.620	1.000
Innate lymphoid cells	0.829	1.09 × 10^−2^	0.658	0.999
CD8^+^ T cells	0.829	1.09 × 10^−2^	0.673	0.984
CD56^hi^ CD16^−^ NK cells	0.821	1.28 × 10^−2^	0.666	0.977
NK cell CD16 MFI	0.814	1.49 × 10^−2^	0.645	0.984
CD16^+^ NK cells	0.800	2.01 × 10^−2^	0.602	0.998
Plasmacytoid dendritic cells	0.796	2.17 × 10^−2^	0.631	0.962
CD25^+^ CD27^+^ CD8^+^ CD45RA^+^ effector memory	0.782	2.89 × 10^−2^	0.553	1.000
MAIT	0.771	3.55 × 10^−2^	0.535	1.000
CD25^+^ CD27^−^ CD8^+^ CD45RA^+^ effector memory	0.764	4.06 × 10^−2^	0.545	0.983
Neutrophil CD16 MFI	0.757	4.64 × 10^−2^	0.528	0.986
CD25^+^ CD8^+^ memory	0.736	6.79 × 10^−2^	0.553	0.919
Conventional dendritic cells	0.736	6.79 × 10^−2^	0.537	0.935
CD21^+^ CD27^+^ Ki67^+^ B cells	0.732	7.21 × 10^−2^	0.478	0.986
CD16^+^ monocytes	0.729	7.66 × 10^−2^	0.539	0.919
Non-classical monocytes/monocytes	0.700	1.21 × 10^−1^	0.465	0.935
CD25^+^ CD8^+^ central memory	0.693	1.35 × 10^−1^	0.466	0.920
CD4^+^ central memory	0.679	1.67 × 10^−1^	0.418	0.939
Neutrophils CD15 MFI	0.675	1.75 × 10^−1^	0.471	0.879
CD8^+^ central memory	0.664	2.03 × 10^−1^	0.453	0.876
CD21^−^ CD27^+^ CD38^lo^ B cells	0.661	2.13 × 10^−1^	0.359	0.963
Intermediate monocytes/monocytes	0.657	2.24 × 10^−1^	0.452	0.862
CXCR5^+^ CD8^+^ transitional memory	0.657	2.24 × 10^−1^	0.448	0.866
CD38^+^ NK cells	0.654	2.34 × 10^−1^	0.406	0.902
CD21^−^CD27^−^CD38^lo^ B cells	0.643	2.68 × 10^−1^	0.395	0.891
CD21^−^ CD27^+^ Ki67^+^ B cells	0.643	2.68 × 10^−1^	0.412	0.874
Monocyte CD16 MFI	0.643	2.68 × 10^−1^	0.398	0.888
Conventional dendritic cells HLA-DR MFI	0.636	2.93 × 10^−1^	0.393	0.878
CXCR5^+^ CD4^+^ memory	0.636	2.93 × 10^−1^	0.425	0.847
Monocyte CD14 MFI	0.636	2.93 × 10^−1^	0.380	0.891
HLA-DR^+^ neutrophils	0.629	3.19 × 10^−1^	0.360	0.897
Non-classical monocytes	0.621	3.47 × 10^−1^	0.371	0.872
Naïve CD8^+^ T cells	0.621	3.47 × 10^−1^	0.407	0.836
CD38^+^ NK cells	0.621	3.47 × 10^−1^	0.406	0.837
CD11c^+^ immature granulocyte	0.614	3.76 × 10^−1^	0.375	0.854
CD38^+^ CD161^+^ NK cells	0.614	3.76 × 10^−1^	0.362	0.867
Intermediate monocytes	0.614	3.76 × 10^−1^	0.392	0.837
CXCR5^+^ CD8^+^ CD45RA^+^ effector memory	0.611	3.91 × 10^−1^	0.404	0.818
CXCR5^+^ CD8^+^ central memory	0.607	4.07 × 10^−1^	0.402	0.812
CD27^−^ HLA-DR^+^ CD8^+^ CD45RA^+^ effector memory	0.607	4.07 × 10^−1^	0.385	0.830
Dendritic cell HLA-DR MFI	0.607	4.07 × 10^−1^	0.340	0.874
CD27^+^ CD8^+^ CD45RA^+^ effector memory	0.604	4.22 × 10^−1^	0.378	0.829
CXCR5^+^ CD8^+^ memory	0.600	4.39 × 10^−1^	0.386	0.814
HLA-DR^+^ CD8^+^ CD45RA^+^ effector memory	0.600	4.39 × 10^−1^	0.383	0.817
CD25^+^ CD8^+^ effector memory	0.600	4.39 × 10^−1^	0.388	0.812
CXCR5^+^ CD4^+^ central memory	0.596	4.55 × 10^−1^	0.385	0.808
CD27^+^ CD8^+^ T cells	0.596	4.55 × 10^−1^	0.389	0.804
CD21^+^ CD27^−^ Ki67^+^ B cells	0.596	4.55 × 10^−1^	0.360	0.833
CD21^−^ CD27^−^ Ki67^+^ B cells	0.593	4.72 × 10^−1^	0.338	0.848
Classical monocytes	0.586	5.07 × 10^−1^	0.348	0.823
CXCR5^+^ CD8^+^ T cells	0.579	5.43 × 10^−1^	0.336	0.821
CD69^+^ CD8^+^ central memory	0.579	5.43 × 10^−1^	0.369	0.788
CD27^−^ CD38^+^ HLA-DR^+^ CD8^+^ CD45RA^+^ effector memory	0.571	5.80 × 10^−1^	0.289	0.854
CD38^+^ HLA-DR^+^ CD8^+^ CD45RA^+^ effector memory	0.564	6.19 × 10^−1^	0.326	0.802
CXCR5^+^ CD4^+^ transitional memory	0.564	6.19 × 10^−1^	0.335	0.794
CD27^−^ CXCR5^+^ CD8^+^ CD45RA^+^ effector memory	0.554	6.78 × 10^−1^	0.315	0.792
CD4^+^ transitional memory	0.550	6.99 × 10^−1^	0.310	0.790
CD8^+^ CD45RA^+^ effector memory	0.550	6.99 × 10^−1^	0.338	0.762
CXCR5^+^ CD8^+^ MAIT	0.550	6.99 × 10^−1^	0.301	0.799
Monocytes	0.543	7.40 × 10^−1^	0.293	0.793
CD8^+^ effector memory	0.543	7.40 × 10^−1^	0.316	0.770
CD69^+^ CD8^+^ transitional memory	0.543	7.40 × 10^−1^	0.331	0.755
CD4^+^ Tregs	0.536	7.82 × 10^−1^	0.303	0.768
CD38^+^ HLA-DR^+^ CD8^+^ MAIT	0.536	7.82 × 10^−1^	0.311	0.761
Ki67^+^ immature granulocyte	0.536	7.82 × 10^−1^	0.266	0.806
Immature granulocyte	0.529	8.25 × 10^−1^	0.253	0.804
HLA-DR^+^ CD8^+^ MAIT	0.521	8.68 × 10^−1^	0.285	0.758
Circulating T-follicular helper	0.521	8.68 × 10^−1^	0.283	0.760
CD69^+^ CD8^+^ effector memory	0.518	8.90 × 10^−1^	0.307	0.729
CD38^+^ HLA-DR^+^ CD8^+^ memory	0.514	9.12 × 10^−1^	0.293	0.736
Naïve CD4^+^ T cells	0.514	9.12 × 10^−1^	0.264	0.764
CD69^+^ CD4^+^ central memory	0.511	9.34 × 10^−1^	0.293	0.729
Eosinophils	0.504	9.78 × 10^−1^	0.233	0.774

*BLR* binary logistic regression probabilities.

Shown *p*-values are corrected for multiple testing using Holm method. Cell populations are expressed as percentages of their respective parent populations except for where mean fluorescence intensity (MFI) is indicated.

**Figure 5:**
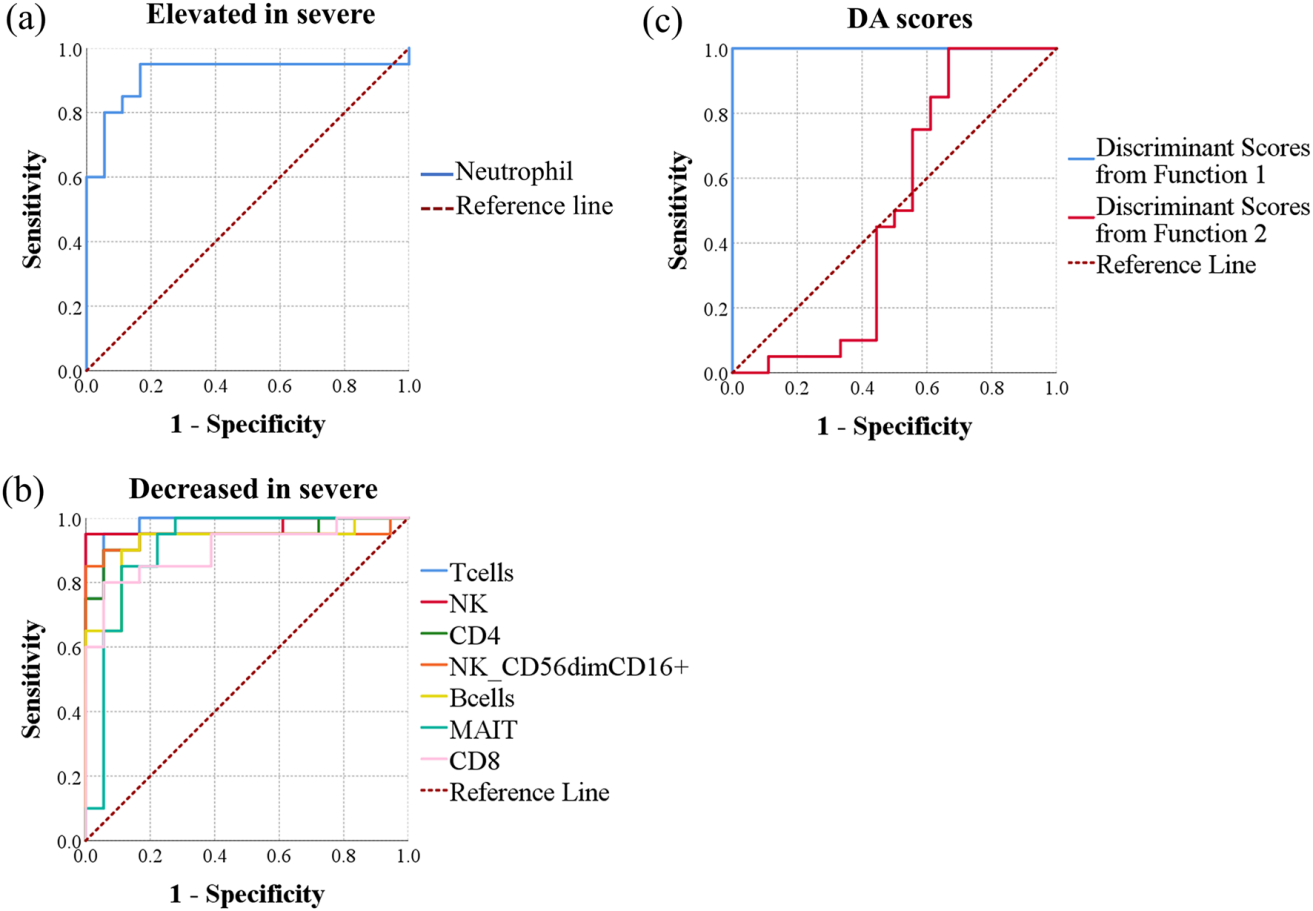
Evaluation of the ability of individual biomarkers and discriminant analysis-based multivariate biomarkers to predict severe COVID-19 disease in a population of healthy donors and COVID-19 patients. ROC curves were generated using data from healthy control participants (n = 12), patients with moderate disease (n = 7), and patients with severe disease (n = 27). ROC curves of (**a**) individual analytes elevated in severe COVID-19, (**b**) individual analytes decreased in severe COVID-19, (**c**) discriminant scores from functions 1 and 2.

Next, we wanted to investigate the fidelity of prediction of severe COVID-19 in a population composed of healthy individuals and COVID-19 patients with either severe or moderate disease. The largest AUCs of individual biomarkers were obtained using T cells, NK cells, CD4+ T cells, CD56dim CD16+ NK cells, B cells, and neutrophils with AUCs of 0.981, 0.969, 0.947, 0.942, 0.922, and 0.917, respectively. A multivariate biomarker based on the discriminant scores of function 1 had a perfect AUC (Fig. 5, Table 6). The separation of groups illustrated in Fig. 1d is compatible with the results obtained, since the severe group is well-separated from the moderate disease group and controls on the first discriminant function, but not the second.

**Table 6:** Estimating the predictive ability of individual biomarkers in identifying severe COVID-19 patients in a population composed of uninfected participants as well as COVID-19 patients with either moderate or severe disease using the area under a receiver operating characteristic curve (AUC) method.

Variable	Area	*p-*value	Asymptotic 95% confidence interval	
			Lower bound	Upper bound
**Combined profiles**				
Discriminant function 1 scores	1.000	1.42 × 10^−7^	1.000	1.000
Discriminant function 2 scores	0.503	0.977	0.295	0.710
**AUC (analytes elevated in severe COVID-19)**				
Neutrophils	0.917	1.16 × 10^−5^	0.812	1.000
Plasmablasts	0.883	5.47 × 10^−5^	0.763	1.000
CD38^+^ CD4^+^ transitional memory	0.875	7.92 × 10^−5^	0.759	0.991
PD1^+^ CD4^+^ transitional memory	0.856	1.82 × 10^−4^	0.724	0.987
PD1^+^ CD8^+^ transitional memory	0.842	3.23 × 10^−4^	0.701	0.982
CD38^+^ CD8^+^ transitional memory	0.839	3.62 × 10^−4^	0.704	0.974
CD27^+^ CD38^+^ CD8^+^ CD45RA^+^ effector memory	0.828	5.61 × 10^−4^	0.692	0.963
PD1^+^ CD4^+^ memory	0.821	7.34 × 10^−4^	0.684	0.957
CD38^+^ CD8^+^ memory	0.819	7.74 × 10^−4^	0.686	0.953
CD38^+^ HLA-DR^+^ CD4^+^ central memory	0.801	1.51 × 10^−3^	0.655	0.947
CD38^+^ HLA-DR^+^ CD8^+^ central memory	0.794	1.94 × 10^−3^	0.654	0.935
CD38^+^ CD4^+^ memory	0.793	2.04 × 10^−3^	0.651	0.935
CD38^+^ CD8^+^ central memory	0.789	2.36 × 10^−3^	0.646	0.932
CD38^+^ HLA-DR^+^ CD4^+^ memory	0.786	2.60 × 10^−3^	0.641	0.932
CD38^+^ HLA-DR^+^ CD4^+^ transitional memory	0.783	2.86 × 10^−3^	0.635	0.931
CD38^+^ CD8^+^ effector memory	0.782	3.00 × 10^−3^	0.634	0.930
PD1^+^ CD8^+^ MAIT	0.781	3.15 × 10^−3^	0.630	0.931
CD38^+^ CD4^+^ effector memory	0.781	3.15 × 10^−3^	0.635	0.926
CD38^+^ HLA-DR^+^ CD8^+^ effector memory	0.772	4.17 × 10^−3^	0.617	0.927
CD38^+^ CD4^+^ central memory	0.769	4.57 × 10^−3^	0.618	0.921
CD38^+^ HLA-DR^+^ CD4^+^ effector memory	0.769	4.57 × 10^−3^	0.617	0.922
PD1^+^ CD8^+^ central memory	0.765	5.24 × 10^−3^	0.613	0.917
PD1^+^ CD4^+^ central memory	0.764	5.48 × 10^−3^	0.611	0.917
CD38^+^ HLA-DR^+^ circulating T-follicular helper	0.760	6.27 × 10^−3^	0.609	0.911
CD38^+^ HLA-DR^+^ CD8^+^ transitional memory	0.760	6.27 × 10^−3^	0.605	0.914
Ki67^+^ CD8^+^ memory	0.758	6.55 × 10^−3^	0.607	0.910
CD38^+^CD8^+^ CD45RA^+^ effector memory	0.758	6.55 × 10^−3^	0.603	0.914
CD27^+^ CD38^+^ HLA-DR^+^ CD8^+^ CD45RA^+^ effector memory	0.757	6.85 × 10^−3^	0.598	0.916
CD38^+^ HLA-DR^+^ CD8^+^ memory	0.756	7.15 × 10^−3^	0.602	0.909
PD1^+^ CD4^+^ effector memory	0.751	8.15 × 10^−3^	0.590	0.913
PD1^+^ CD8^+^ memory	0.744	1.01 × 10^−2^	0.588	0.901
Ki67^+^ CD8^+^ central memory	0.736	1.30 × 10^−2^	0.579	0.894
CD38^+^ CD8^+^ MAIT	0.729	1.59 × 10^−2^	0.556	0.902
Ki67^+^ CD8^+^ effector memory	0.725	1.79 × 10^−2^	0.564	0.886
CD27^+^ Ki67^+^ CD8^+^ CD45RA^+^ effector memory	0.725	1.79 × 10^−2^	0.562	0.888
CD38^+^ HLA-DR^+^ CD8^+^ CD45RA^+^ effector memory	0.725	1.79 × 10^−2^	0.558	0.892
HLA-DR^+^ CD4^+^ central memory	0.725	1.79 × 10^−2^	0.555	0.895
Ki67^+^ CD8^+^ MAIT	0.708	2.83 × 10^−2^	0.540	0.877
HLA-DR^+^ CD4^+^ memory	0.706	3.05 × 10^−2^	0.528	0.883
HLA-DR^+^ circulating T-follicular helper	0.703	3.28 × 10^−2^	0.535	0.870
CD27^−^ CD38^+^ CD8^+^ CD45RA^+^ effector memory	0.703	3.28 × 10^−2^	0.534	0.872
Eosinophils	0.697	3.79 × 10^−2^	0.524	0.870
Ki67^+^ CD4^+^ memory	0.690	4.52 × 10^−2^	0.518	0.862
HLA-DR^+^ CD8^+^ central memory	0.683	5.37 × 10^−2^	0.502	0.864
CD27^−^ CD38^+^ HLA-DR^+^ CD8^+^ CD45RA^+^ effector memory	0.683	5.37 × 10^−2^	0.506	0.860
CD27^−^ Ki67^+^ CD8^+^ CD45RA^+^ effector memory	0.678	6.13 × 10^−2^	0.506	0.849
HLA-DR^+^ CD8^+^ memory	0.676	6.34 × 10^−2^	0.500	0.852
PD1^+^ CD8^+^ CD45RA^+^ effector memory	0.675	6.55 × 10^−2^	0.503	0.847
Ki67^+^ CD8^+^ CD45RA^+^ effector memory	0.669	7.45 × 10^−2^	0.496	0.843
Ki67^+^ CD4^+^ central memory	0.669	7.45 × 10^−2^	0.493	0.845
CD69^+^ CD8^+^ MAIT	0.667	7.94 × 10^−2^	0.490	0.843
HLA-DR^+^ CD8^+^ effector memory	0.667	7.94 × 10^−2^	0.489	0.844
CD69^+^ CD4^+^ effector memory	0.664	8.45 × 10^−2^	0.485	0.843
PD1^+^ CD8^+^ effector memory	0.663	8.72 × 10^−2^	0.488	0.837
HLA-DR^+^ CD4^+^ effector memory	0.660	9.28 × 10^−2^	0.481	0.838
Ki67^+^ CD8^+^ transitional memory	0.658	9.56 × 10^−2^	0.484	0.833
HLA-DR^+^ CD8^+^ MAIT	0.658	9.56 × 10^−2^	0.480	0.837
CD27^+^ HLA-DR^+^ CD8^+^ CD45RA^+^ effector memory	0.657	9.86 × 10^−2^	0.476	0.838
CD69^+^ CD4^+^ transitional memory	0.651	1.11 × 10^−1^	0.476	0.827
Ki67^+^ CD4^+^ effector memory	0.643	1.32 × 10^−1^	0.466	0.820
HLA-DR^+^ CD8^+^ transitional memory	0.642	1.36 × 10^−1^	0.458	0.825
CD27^+^ CD69^+^ CD8^+^ CD45RA^+^ effector memory	0.640	1.40 × 10^−1^	0.455	0.826
CD21^−^ CD27^−^ CD38^lo^ B cells	0.639	1.44 × 10^−1^	0.456	0.821
CD38^+^ HLA-DR^+^ CD8^+^ MAIT	0.635	1.56 × 10^−1^	0.450	0.820
CD27^−^ PD1^+^ CD8^+^ CD45RA^+^ effector memory	0.633	1.61 × 10^−1^	0.452	0.814
HLA-DR^+^ CD4^+^ transitional memory	0.622	1.98 × 10^−1^	0.438	0.807
CXCR5^+^ CD8^+^ effector memory	0.618	2.14 × 10^−1^	0.430	0.806
CD27^−^ CD8^+^ CD45RA^+^ effector memory	0.617	2.19 × 10^−1^	0.426	0.807
CD27^−^ CD8^+^ T cells	0.614	2.31 × 10^−1^	0.428	0.799
CD69^+^ CD4^+^ memory	0.611	2.42 × 10^−1^	0.427	0.795
Classical monocytes/monocytes	0.608	2.54 × 10^−1^	0.424	0.792
Immature granulocyte	0.607	2.60 × 10^−1^	0.423	0.791
CD4^+^ CD45RA^+^ effector memory	0.607	2.60 × 10^−1^	0.425	0.789
Ki67^+^ CD4^+^ transitional memory	0.604	2.73 × 10^−1^	0.420	0.788
HLA-DR^+^ CD8^+^ CD45RA^+^ effector memory	0.603	2.79 × 10^−1^	0.418	0.787
CD27^−^ HLA-DR^+^ CD8^+^ CD45RA^+^ effector memory	0.597	3.06 × 10^−1^	0.413	0.781
CD25^+^ NK cells	0.588	3.57 × 10^−1^	0.402	0.773
Plasmacytoid dendritic cell HLA-DR MFI	0.582	3.88 × 10^−1^	0.395	0.769
CD25^+^ CD8^+^ effector memory	0.581	3.97 × 10^−1^	0.393	0.768
CD4^+^ effector memory	0.581	3.97 × 10^−1^	0.395	0.766
Monocytes	0.578	4.13 × 10^−1^	0.387	0.768
CD27^−^ CD69^+^ CD8^+^ CD45RA^+^ effector memory	0.576	4.21 × 10^−1^	0.389	0.764
CD27^+^ NK cells	0.572	4.47 × 10^−1^	0.381	0.763
Immature granulocyte CD16 MFI	0.569	4.65 × 10^−1^	0.384	0.755
CD69^+^ CD8^+^ CD45RA^+^ effector memory	0.563	5.11 × 10^−1^	0.369	0.756
PD1^+^ NK cells	0.556	5.59 × 10^−1^	0.368	0.743
Classical monocytes	0.551	5.89 × 10^−1^	0.364	0.739
CD25^+^ CD8^+^ central memory	0.544	6.40 × 10^−1^	0.357	0.732
CD123^+^ immature granulocyte	0.542	6.61 × 10^−1^	0.355	0.728
CD8^+^ CD45RA^+^ effector memory	0.542	6.61 × 10^−1^	0.354	0.730
CXCR5^+^ CD4^+^ transitional memory	0.539	6.82 × 10^−1^	0.351	0.726
CD21^−^ CD27^+^ CD38^lo^ B cells	0.536	7.04 × 10^−1^	0.342	0.730
Intermediate monocytes	0.526	7.81 × 10^−1^	0.338	0.714
CXCR5^+^ CD4^+^ effector memory	0.525	7.92 × 10^−1^	0.334	0.716
CD25^+^ CD8^+^ memory	0.524	8.04 × 10^−1^	0.337	0.710
CD4^+^ Tregs	0.519	8.38 × 10^−1^	0.330	0.709
Ki67^+^ NK cells	0.517	8.61 × 10^−1^	0.328	0.706
Eosinophil CD15 MFI	0.514	8.84 × 10^−1^	0.326	0.702
Ki67^+^ neutrophils	0.506	9.53 × 10^−1^	0.318	0.693
CD161^+^ monocytes	0.504	9.65 × 10^−1^	0.308	0.700
CD16^+^ immature granulocyte	0.503	9.77 × 10^−1^	0.316	0.690
HLA-DR^+^ NK cells	0.503	9.77 × 10^−1^	0.316	0.689
Circulating T-follicular helper	0.500	1.00	0.312	0.688
B cells HLA-DR MFI	0.500	1.00	0.306	0.694
**AUC (analytes decreased in severe COVID-19)**				
T cells	0.981	4.24 × 10^−7^	0.946	1.000
NK cells	0.969	7.78 × 10^−7^	0.909	1.000
CD4^+^ T cells	0.947	2.52 × 10^−6^	0.871	1.000
CD56^dim^ CD16^+^ NK cells	0.942	3.35 × 10^−6^	0.849	1.000
B cells	0.922	8.84 × 10^−6^	0.829	1.000
MAIT	0.911	1.51 × 10^−5^	0.805	1.000
CD8^+^ T cells	0.903	2.24 × 10^−5^	0.804	1.000
Innate lymphoid cells	0.896	3.10 × 10^−5^	0.798	0.994
NK cells CD16 MFI	0.864	1.28 × 10^−4^	0.749	0.978
CD56^hi^ CD16^−^ NK cells	0.858	1.62 × 10^−4^	0.735	0.982
Neutrophil CD16 MFI	0.850	2.30 × 10^−4^	0.715	0.985
Monocyte HLA-DR MFI	0.833	4.51 × 10^−4^	0.686	0.981
Dendritic cells	0.808	1.17 × 10^−3^	0.664	0.952
Plasmacytoid dendritic cells	0.781	3.15 × 10^−3^	0.627	0.934
CD16^+^ NK cells	0.767	5.01 × 10^−3^	0.611	0.923
Conventional dendritic cells	0.753	7.80 × 10^−3^	0.595	0.911
CD38^+^ NK cells	0.749	8.88 × 10^−3^	0.593	0.904
Neutrophil CD15 MFI	0.724	1.86 × 10^−2^	0.563	0.885
CD25^+^ CD8^+^ MAIT	0.722	1.93 × 10^−2^	0.559	0.886
Non-classical monocytes/Monocytes	0.708	2.83 × 10^−2^	0.540	0.877
CD21^+^ CD27^+^ CD38^lo^ B cells	0.701	3.40 × 10^−2^	0.528	0.875
CD21^+^ CD27^+^ Ki67^+^ B cells	0.682	5.55 × 10^−2^	0.507	0.857
Monocyte CD16 MFI	0.681	5.74 × 10^−2^	0.509	0.853
Monocyte CD14 MFI	0.678	6.13 × 10^−2^	0.502	0.854
CD16^+^ monocytes	0.678	6.13 × 10^−2^	0.504	0.851
CD21^−^ CD27^+^ Ki67^+^ B cells	0.671	7.22 × 10^−2^	0.495	0.846
CD25^+^ CD8^+^ CD45RA^+^ effector memory	0.669	7.45 × 10^−2^	0.491	0.848
CXCR5^+^ CD8^+^ central memory	0.631	1.69 × 10^−1^	0.445	0.816
CD69^+^ CD8^+^ central memory	0.622	1.98 × 10^−1^	0.429	0.815
Naïve CD8^+^ T cells	0.614	2.31 × 10^−1^	0.427	0.801
CD11c^+^ immature granulocyte	0.614	2.31 × 10^−1^	0.432	0.796
Dendritic cells HLA-DR MFI	0.611	2.42 × 10^−1^	0.414	0.808
CD69^+^ CD8^+^ transitional memory	0.611	2.42 × 10^−1^	0.414	0.809
CD38^+^ NK cells	0.608	2.54 × 10^−1^	0.420	0.796
CD25^+^ CD27^−^ CD8^+^ CD45RA^+^ effector memory	0.608	2.54 × 10^−1^	0.425	0.791
CXCR5^+^ CD4^+^ T cells	0.604	2.73 × 10^−1^	0.415	0.793
CD21^+^ CD27^−^ Ki67^+^ B cells	0.604	2.73 × 10^−1^	0.422	0.787
Conventional dendritic cell HLA-DR MFI	0.604	2.73 × 10^−1^	0.416	0.793
CXCR5^+^ CD8^+^ transitional memory	0.601	2.86 × 10^−1^	0.412	0.791
CXCR5^+^ CD8^+^ CD45RA^+^ effector memory_	0.597	3.06 × 10^−1^	0.411	0.784
CXCR5^+^ CD4^+^ central memory_	0.588	3.57 × 10^−1^	0.400	0.775
CD38^+^ HLA-DR^+^ NK cells	0.586	3.65 × 10^−1^	0.399	0.774
CD8^+^ central memory	0.582	3.88 × 10^−1^	0.396	0.768
Naïve CD4^+^ T cells	0.579	4.05 × 10^−1^	0.393	0.766
HLA-DR^+^ neutrophils	0.579	4.05 × 10^−1^	0.396	0.762
CD38^+^ CD161^+^ NK cells	0.572	4.47 × 10^−1^	0.380	0.764
CD8^+^ effector memory	0.567	4.83 × 10^−1^	0.380	0.753
CD8^+^ transitional memory	0.567	4.83 × 10^−1^	0.381	0.753
CXCR5^+^ CD8^+^ memory T cells	0.565	4.92 × 10^−1^	0.372	0.758
CD27^+^ CD8^+^ CD45RA^+^ effector memory	0.565	4.92 × 10^−1^	0.375	0.755
CXCR5^+^ CD8^+^ T cells	0.564	5.01 × 10^−1^	0.371	0.756
CD69^+^ CD8^+^ effector memory	0.560	5.30 × 10^−1^	0.362	0.758
Ki67^+^ immature granulocyte	0.560	5.30 × 10^−1^	0.373	0.746
CXCR5^+^ CD8^+^ MAIT	0.557	5.49 × 10^−1^	0.361	0.753
CD25^+^ CD27^+^ CD8^+^ CD45RA^+^ effector memory_	0.550	5.99 × 10^−1^	0.361	0.739
CD4^+^ central memory	0.544	6.40 × 10^−1^	0.353	0.736
CD25^+^ CD8^+^ transitional memory	0.539	6.82 × 10^−1^	0.348	0.729
Non-classical monocytes	0.539	6.82 × 10^−1^	0.348	0.730
Intermediate monocytes/monocytes	0.536	7.04 × 10^−1^	0.349	0.723
CD21^+^ CD27^−^ CD38^lo^ B cells	0.531	7.48 × 10^−1^	0.339	0.722
CD27^+^ CD8^+^ T cells	0.526	7.81 × 10^−1^	0.336	0.717
CD27^+^ CXCR5^+^ CD8^+^ CD45RA^+^ effector memory	0.521	8.26 × 10^−1^	0.328	0.714
CD69^+^ CD8^+^ memory	0.517	8.61 × 10^−1^	0.319	0.714
CD21^−^ CD27^−^ Ki67^+^ B cells	0.515	8.72 × 10^−1^	0.324	0.706
CD4^+^ transitional memory	0.508	9.30 × 10^−1^	0.321	0.696
CD69^+^ CD4^+^ central memory	0.507	9.42 × 10^−1^	0.315	0.698
CD27^−^ CXCR5^+^ CD8^+^ CD45RA^+^ effector memory	0.504	9.65 × 10^−1^	0.318	0.690

Shown *p*-values are corrected for multiple testing using Holm method. Cell populations are expressed as percentages of their respective parent populations except for where mean fluorescence intensity (MFI) is indicated.

Finally, we tested the predictive ability of our models by employing them to classify eight participants by a blinded investigator. These participants included two with mild, four with moderate, and two with severe disease. There were no uninfected controls. Please note that there was no mild group in any of the models we constructed, but we had data for these two patients with mild disease and thought to include them to see how they would be classified. Using the discriminant scores of Model 1, one of the mild patients was classified as uninfected control, while the other was classified as moderate disease. Only one of the moderate patients was correctly classified, while the other three as well as the two severe patients were classified as severe. Counting the classification of mild patient as moderate correct (due to the absence of a mild category in the model), the overall RCC was 50% (Table 7). Models 2 and 2′ correctly classified seven participants, while a moderate participant and a mild participant were misclassified as control by models 2 and 2′, respectively. Both models showed an RCC of 87.5% (Table 7). The two participants with mild disease were classified as moderate by both models 3 and 3′. Both patients with severe disease were correctly classified by model 3, while model 3′ misclassified one of them as moderate. For participants with moderate disease, only one of them was classified as such by model 3—the remaining three were misclassified as severe—and model 3′ correctly classified all four participants (Table 7).

**Table 7:** Blinded classification of unknown participants.

Participant ID	Actual classification	Model 1	Model 2	Model 3	Model 2′	Model 3′
U1	Moderate	Severe	Covid	Severe	Covid	Moderate
U2	Moderate	Moderate	Control	Severe	Covid	Moderate
U3	Severe	Severe	Covid	Severe	Covid	Severe
U5	Severe	Severe	Covid	Severe	Covid	Moderate
U6	Moderate	Severe	Covid	Moderate	Covid	Moderate
U7	Moderate	Severe	Covid	Severe	Covid	Moderate
U8	Mild	Moderate	Covid	Moderate	Covid	Moderate
U9	Mild	Control	Covid	Moderate	Control	Moderate
Overall RCC	–	50%	87.5%	62.5%	87.5%	87.5%

*RCC* rate of correct classification.

## Discussion

The current study uses immune profiles to distinguish between severe and moderate COVID-19 patients, and between COVID-19 patients and uninfected control participants. The RCCs, a measure of the fidelity of prediction, ranged from a modest 70 to 100%. Fidelity of prediction differed by the number (i.e., two or three groups) and identities (i.e., moderate and severe COVID-19 and uninfected controls) of the groups being distinguished from each other, as well as whether DA or BLR was used. Our RCCs are comparable to those obtained in previous studies. Mueller and coworkers used BLR to predict immunophenotypes that correlated with COVID-19 disease severity with RCCs of 80–83%^14^.

The original analysis of our data published by Kuri-Cervantes in 2020 identified COVID-19-specifc and severe disease-specific changes consistent with other groups’ findings^8^. The reader is encouraged to review said publication for detailed description of the findings. From Kuri-Cervantes work and the work of others, we learned that compared to uninfected persons, severe COVID-19 is characterized by lower frequencies of lymphocytes^8,15^, total B cells^8^, total T cells^8^, CD4^+^ T cells^8^, CD8^+^ T cells^8^, CD8^+^ MAIT cells^8^, ILCs^8^, and NK cells^8^, as well as increased frequencies of neutrophils^15,19^ and monocytes^8,15^, and higher neutrophil-to-lymphocyte ratio. Neutrophil activation has also been implicated in severe COVID-19^19^. Also, dendritic cell depletion and dysfunction have previously been linked to severe COVID-19 disease^36^.

From all models of the current study, we conclude that severe COVID-19 is best characterized by depletion of NK cells and T cells. Model 3/3′ of the current study showed that the most characteristic features of severe COVID-19 that set it apart from moderate disease were low frequencies of NK cells and actSMB, down-regulation of monocyte HLA-DR, and increased frequency of neutrophils. From model 1/1′, we learned that increased frequencies of actSMB and actNeut were the most prominent features of moderate disease, setting it apart from both severe disease and healthy controls. This pattern is consistent with a prominent role of NK cells, actSMB, and actNeut in steering the course of COVID-19 toward milder disease and better prognosis. We also show that the most prominent feature of COVID-19, including moderate and severe disease, that sets it apart from healthy controls was MAIT, highlighting the function of this population of immune cells and its relevance to COVID-19.

BLR was generally superior to DA in achieving higher RCCs. DA functions optimally with the least amount of error when all relevant assumptions are satisfied^37^. These assumptions were not fully satisfied in our dataset, with most V_i_s deviating from normal Gaussian distribution and some level of multicollinearity, heteroscedasticity, and putative outliers present. It is not clear how these issues affect our results and the conclusions we derive from them. Early research from the 1960s and 1970s indicated that DA can function satisfactorily with non-normal data under certain conditions and with some, but not all, forms of non-normality^38^. Some of these early studies concluded that DA performs poorly when analyzing non-normally-distributed data, but these studies derived their conclusions from experiments that dealt with drastic levels of skewness and kurtosis^38^. In a recent study by Zuber and Tata, a high level of error was observed while using DA to analyze non-normal data^39^; the dataset used by these authors was also an extreme case of non-normality (data not shown). Lantz showed that error decreased as sample size increased, and that error increased with increasing deviation from normal distribution, the degree of heteroscedasticity, and the number of variables^40^. The Lantz work is important because it demonstrated gradients of negative impacts exerted by a number of isolated “anomalies” on the performance of DA. Non-normal distribution of empirical data is not uncommon in health sciences as well as other fields of knowledge^41^. In practice, this problem has been historically and broadly ignored^37^. Some investigators suggested resolving the problem of non-normality by applying a transformation (e.g., log transformation), but doing so may itself lead to erroneous conclusions by altering the interrelationships among observations and variables^37^. Although eliminating non-normality in biomedical research is mostly unpractical, there are indicators that doing so may not always be necessary. In our previous studies, DA performed well despite a lack of normal distribution^42^.

A limitation of the current study is the relatively small sample size. Sample size requirement in DA and similar techniques is not well defined. Based on currently available data, it has been suggested that the size of the smallest group in a dataset should outnumber the independent variables by at least three fold^43^. Another issue is the proportional size of groups. When the training dataset is severely unbalanced (i.e., group sizes are very different), higher RCCs tend to occur in larger groups, while the converse is true for smaller groups^44^. This phenomenon was observed in our data, for example, in model 1, the RCC of the largest group (severe) was higher than the RCC of the intermediate size group (control), and the RCCs of both groups were higher than the RCC of the smallest group (moderate). The same was observed in model 2 and the BLR model 3′. It might be worth noting that these were all the models in which this trend could be identified since both remaining models had 100% RCCs. Therefore, all available data agree that sample size is likely an important criterion. Although reaching a sample size that satisfies the three-fold role mentioned above is frequently not achievable—due to cost and technical limitations—in biomedical research, one should at least repeat experiments multiple times with different sample sizes and confirm the consistency of obtained results.

Furthermore, both non-normal distribution and sample size affect the reliability of Box’s M test, which tests homoscedasticity of the data. One of the disadvantages of this test is that it was originally designed for use with normally distributed data^25^ and it, therefore, lacks robustness even with mild deviations from normality^26^. It is also problematic when the sample size is either too large or too small. Box’s M test tends to suggest a significant lack of homoscedasticity (i.e., *p*-value below the threshold of significance) when the sample size is too large, even in the presence of acceptable levels of variance homogeneity. This issue could be overcome by using a more stringent threshold than the usual alpha of 0.05 (e.g., 0.001)^45^. Box’s M test lacks statistical power with small sample sizes^46,47^, and tends to falsely suggest data homoscedasticity (i.e., *p*-value above the threshold of significance) even when the level of heteroscedasticity is problematic^43^. In our case, Box’s M suggested significant heteroscedasticity, which is not unexpected given the profound deviation from Gaussian distribution in our data. Therefore, we relied on the non-parametric Levene’s test, which asserted the homogeneity of variance–covariance matrices between groups.

Due to the difficulty in fully satisfying the assumptions required for optimal performance of the linear discriminant function, further research precisely defining the exact limitations of DA in the presence of non-normality in real-life data and the practical implications for health and other sciences is needed. In the absence of such guidelines that definitively delineate when or when not to use DA and similar techniques, the investigator is forced to choose between abandoning these techniques all together or cautiously using them hoping to reach useful conclusions in an admittedly suboptimal scenario. Another interesting possibility is running two or more techniques (e.g., DA and BLR) in parallel, hoping to have matching results. This latter approach is only valid, however, when the mathematical basis of the techniques used are dissimilar enough that the methods could be considered independent.

For future development of this project, we hope to be able to test on models in patients to establish whether they are effective at classifying patients as severe or moderate disease. By testing a wide range of biomarkers in a group of COVID-19 patients, as was done in Kuri-Cervantes’ work, and stratifying those patients into groups of potential disease severity, we hope to demonstrate the clinical usefulness of our model as a predictor of disease course. Further, we hope to update these models with more data to establish more effective distinguishing parameters between the different groups of disease severity. Using updated models with more patient data, we hope to gain additional insights into the pathogenesis of COVID-19 and what determines disease course. Finally, we are interested in updating these models with data involving the cytokine response to COVID-19 infection, to see if levels of specific cytokines can help to distinguish severity of COVID-19 infection.

Overall, we conclude that DA remains an invaluable dimension reduction and classification technique in health sciences, but we encourage careful interpretation of results and thorough consideration of the level of deviation from the assumptions of DA and the level of congruence of conclusion derived from DA with other methods, such as logistic regression. We show that the most prominent immunological hallmarks of COVID-19 disease include depletion of NK cells and T cells and hyperactivation of neutrophils and class-switched memory B cells. We also show that the most characteristic early immunological markers of severe COVID-19 when compared to moderate disease include a more severe depletion of NK cells, depletion of actSMB cells, an impaired activation of monocytes, and relative expansion of neutrophils. The pathophysiology of COVID-19, including severe or moderate disease, involves depletion of CD8^+^ MAIT cells, a fact that could be exploited in future studies to develop a better understanding of disease pathogenesis or develop interventional novel strategies. Further analyses are needed to define the most important biomarkers out of all measurable, relevant analytes (cell populations, expression of surface proteins, cytokines, and more) and the optimum modeling methods that maximizes the fidelity of disease severity prediction.

## Methods

### Source of data

The current study is a reanalysis of previously published data^8^. We analyzed 27 COVID-19 patients (7 moderate disease and 20 severe disease) and 11 healthy control subjects. Data were downloaded from the website of the Human Pancreas Analysis Program (HPAP; https://hpap.pmacs.upenn.edu), Perelman School of Medicine, University of Pennsylvania, Philadelphia, PA. Blood specimens were collected and analyzed from 8 additional participants of unknown disease status at the Perelman School of Medicine and blinded data were shared with the University of Missouri Kansas City team for analysis. Informed consent was obtained from all participants or their surrogates, and the project was approved by University of Pennsylvania ethical research board. The study was conducted in Declaration of Helsinki. Flow cytometry data analysis was performed using FlowJo™ Software^48^. One hundred and seventy-one flow cytometry variables (Supplemental Table S1) were selected for inclusion in this study.

### Initial screening of data

DA functions optimally when certain assumptions are satisfied. The data are assumed to be multivariate normal^38^, which requires univariate normality of each of the variables^49^. Furthermore, it has been shown that linear combinations of two or more normally distributed continuous variables are also normally distributed^50^. Therefore, we tested the normality of each of the variables individually using the Shapiro–Wilk’s test. The null hypothesis tested by the Shapiro–Wilk’s test is that a dataset does not significantly differ from a normal distribution. A *p*-value is computed to reject or retain the null hypothesis. A statistic (*W*) is computed that equals “1” for datasets that perfectly conform to normality, while smaller values imply proportionate deviations from normal distribution. Therefore, a dataset is considered normally distributed when *W* approaches “1” and the null hypothesis is retained by a *p*-value greater than 0.05^51^. We also computed skewness (i.e., asymmetric distribution around the mean) and kurtosis (i.e., the sharpness of the frequency-distribution curve) for each predictor variable. The range of skewness and kurtosis values within which data are considered normal is not definitively identified in the literature. Multiple investigators accept skewness and kurtosis values between − 1 and + 1^52^, while others accept a wider range from − 2 to + 2^53^. Another assumption of DA is the absence of multicollinearity or highly correlated predictor variables^38^. Pearson moment correlation coefficient was used to calculate a correlation matrix including all predictor variables to verify the absence of highly correlated variables with correlation coefficients approaching 1 or − 1. DA also assumes homoscedasticity or equality of variance–covariance matrices across all levels of the dependent variable^54^. This can be tested in SPSS using Box’s M test, which tests the null hypothesis stipulating that the variance–covariance matrices are equal across all groups^25^. However, given that most of our variables were not normally distributed (Supplemental Table S1), the nonparametric Levene’s test—a more robust test when the assumption of multivariate normality is violated^55^—was more appropriate. Both tests were performed to compare the results and determine whether conclusions made based on one test were consistent with those made based on the other. Shapiro–Wilk’s test, Pearson correlations, Box’s M test, and nonparametric Levene’s test were performed using IBM SPSS version 26 (IBM Corporation, Armonk, NY). We also screened the data for the presence of potential outliers, which is another assumption of DA^56^. We used a modification of the method described by Hoaglin et al.^57^. Briefly, scores outside a range defined by lower and upper limits were considered potential outliers. The lower and upper limits were calculated using Eqs. (1) and (2), respectively. Quartiles were determined using Excel function “QUARTILE.EXC”.

1 $${\text{Lower limit }} = {\text{ Q1}}{-}k\left( {{\text{IQR}}} \right),$$

2 $${\text{Upper limit }} = {\text{ Q3}}^{ + } k\left( {{\text{IQR}}} \right),$$

where Q1 and Q3 are the first and third quartiles, IQR is the interquartile range obtained by subtracting Q1 from Q3, and *k* is a constant equal to 2.2^57^.

### Discriminant analysis

DA is a data reduction method that combines correlated predictor variables into fewer new variables called canonical discriminant functions. The goal of DA is to simplify visualization and interpretation of the data, while maximizing discrimination between groups of interest. DA can be performed by sequentially incorporating predictor variables that significantly improve the discriminant model, while ignoring variables that offer no significant improvement to the model; this method is called stepwise DA. DA can also be done by incorporating all variables at once. In this study, we used the stepwise method to limit the discriminant model to the most effective predictor variables. The overall predictive ability and significance of the discriminant model were evaluated by the Wilks’ λ statistic, which reflects the proportion of variance in the discriminant model that is not predictive of group membership. Wilks’ λ ranges from zero to one, with zero corresponding to perfect prediction of group membership and one corresponding to a complete lack of group predictive power. A Chi-square test was performed to test the null hypothesis that the discriminant model’s predictive power is no different from random prediction with a *p*-values < 0.05 indicating that the model is significantly different from random prediction^43^. Discriminant models were also evaluated by classifying subjects into groups based on the model and computing the rate of correct classification (RCC). Each subject was removed from the model prior to classification into a group. We have also evaluated the effectiveness of individual discriminant functions and the relative importance of each variable included in the model. Discriminant functions were evaluated based on the corresponding eigenvalue and canonical correlation. The eigenvalue reflects the amount of variance explained by the discriminant function, thus, the greater this value, the better the quality of the discriminant function^58^. Canonical correlations measure the discriminant function’s correlation with the groups, which is higher for higher quality functions^59^. One way we evaluated the potential of individual variables for being beneficial for the model was by performing one-way ANOVA to test differences between group means among all variables, regardless of whether they were incorporated into the model, with an adjusted *p*-value threshold of 0.05. The *p*-values were adjusted for multiple testing using the method described by Holm^60^, which was executed in SPSS using a modified version of the syntax written by Raynald Levesque and improved by Marta Garcia-Granero^61^. We also performed pairwise comparisons on the variables incorporated into a model using a t-test *p*-value cutoff of 0.05 with a Holm–Sidak correction for multiple testing. This was done using GraphPad Prism version 6 for Windows (GraphPad Software, San Diego, California USA, www.graphpad.com). Individual variables were also evaluated using the Wilks’ λ statistic, which reflects the proportion of the biomarker variance that was not explained by differences between groups. Wilks’ λ of the most useful variables to the discriminant model tend approach zero, implying that almost all variance of that variable can be explained by differences between groups^43^. The third criterion looked at to evaluate individual variables was the direct contribution of the variable to the discriminant model expressed as a scaler or the standardized canonical discriminant function coefficient^62^. DA was performed using IBM SPSS version 26 (IBM Corporation, Armonk, NY).

### Binary logistic regression

Binary logistic regression was performed using IBM SPSS version 26 (IBM Corporation, Armonk, NY). This statistical technique uses a participant’s scores on one or more predictor variables to predict the odds of that participant falling in one of the two outcomes of a binary dependent variable^43^. For example, using binary logistic regression, we can calculate the odds of survival of a patient based on the patient’s clinical and demographic data. Since the odds and predictor variables rarely form linear relationships, the natural logarithm of odds—also known as Logit or *L*_*i*_—is computed from the scores of predictor variables (*X*_*i*_) multiplied times weights or coefficients (*B*_*i*_) (Eq. 3). These coefficients are selected to maximize the goodness of fit of the model. The coefficients selected are the ones that lead to the highest success rate in correctly classifying participants into their corresponding groups. These weights represent the predicted change in *L*_*i*_ for each unit increase in the corresponding predictor variable, therefore, they can be used to evaluate the importance of individual predictor variables to the model.

3 $${\text{L}}_{\text{i}} \, = {B}_{0} + {B}_{1}{X}_{1 }+{ B}_{2}{X}_{2} + \cdots + {B}_{n}{X}_{n},$$

where *L*_*i*_ is the Logit statistic, *B*_*i*_ is the *i*th logistic regression coefficient, and *X*_*i*_ is the *i*th predictor variable.

Since *L*_*i*_ is the natural log of odds, it can be used to calculate the odds of belonging to a target group or the probability of belonging to that group. The odds can be calculated by simply raising *e* to the power of *L*_*i*_ (Eq. 4), and the probability (*Y*_*i*_) can be calculating by substituting into Eq. (5).

4 $${\text{Odds}} \, = {e}^{{L}_{i}} = {e}^{{B}_{0} + \, {B}_{1}{X}_{1 \, }+{ B}_{2}{X}_{2} + \cdots + \, {B}_{n}{X}_{n}},$$

5 $${\text{Yi}} = \frac{{e}^{Li}}{1 + \, {e}^{Li}},$$

where *Y*_*i*_ is the probability of belonging to a target group, *L*_*i*_ is the Logit statistic, and *e* is the base of the natural log and is approximately equal to 2.71828.

Here, we developed models based on participants with known groups, hoping to ultimately enable the use of these models in clinical practice to predict patient outcomes. The quality of logistic regression models was evaluated based on multiple criteria. One criterion is how different the predictive model is from a “null” model that contains no predictor variables. A null model is based on the number of participants in each of the two groups with no predictors in the equation. By substitution in Eq. (5), *L*_*i*_ of a null model is equal to *B*_*0*_, which is also equal to ln [Odds]. In the absence of predictors, the odds of having one outcome in a null model is obtained by dividing the number of times that outcome occurs by the number of times the alternate outcome occurs (Eq. 6)^43^.

6 $${\text{Odds}}=\frac{Number \,of\, participants\, in\, the \,target\, group}{Number \,of \,participants\, in \,the\, alternate\, group}.$$

A null model assigns the same odds to all participants and predicts them all to belong to one group, the one with larger number of participants. Naturally, many participants will be misclassified using the null model. Predictive models must be significantly better at correctly classifying participants compared to the null model. Since we built our models stepwise, the first step had to significantly improve the fidelity of prediction over the null model and each subsequent step had to introduce a significant improvement over its predecessor. The model is complete when no more significant improvements can be made by incorporating additional predictors or when all predictors have been incorporated. The significance of the difference between a predictive model and the null model is tested by a Chi-square test with *p*-values below 0.05 considered significance. Once a predictive model is determined to be a significant improvement over the null model, additional testing is needed to evaluate the quality of this improvement. For this this purpose, we used the Hosmer–Lemeshow test and the Nagelkerke’s pseudo-*R*^2^. The Hosmer–Lemeshow test tests the null hypothesis that the model predicts group membership with perfect accuracy. This null hypothesis is retained with *p*-values greater than 0.05 when group membership predicted by the logistic regression model match observed group membership^63^. Nagelkerke’s pseudo-*R*^2^ can take values between zero and one, with higher values obtained with better models^64^. Finally, we empirically evaluated binary logistic regression models by calculating the RCC associated with each model. For high quality models, it may also be informative to evaluate the relative contribution of predictor variables to the model. This was evaluated by testing the significance of each variable’s contribution and the regression coefficients assigned to each predictor variable. The regression coefficients reflect the predicted change in log odds with unit change in the predictor variable, with odds here referring to the odds of falling into a target group^43^. The limitation of this approach is that it fails to compute regression coefficients or a meaningful *p*-value in datasets with complete or quasi-complete (i.e. near complete) group separation^65^.

### Receiver operating characteristic curve (ROC) and area under the curve (AUC)

ROC/AUC analyses were performed using IBM SPSS version 26 (IBM Corporation, Armonk, NY). The ROC curve is generated by plotting the rate of true positives (sensitivity) against the rate of false positives (1 − specificity) for all possible threshold values. For a test with 100% sensitivity and specificity, the AUC is equal to 1, while a useless test has an AUC of 0.5. Asymptotic significance of the AUC is evaluated by testing the null hypothesis stating that the test has an AUC of 0.5. The null hypothesis is rejected when the adjusted *p*-value is lower than 0.5. Adjusting for multiple testing was performed using Holm method.

## Supplementary Information

Supplementary Legends.

Supplementary Table S1.

Supplementary Table S2.

Supplementary Table S3.

Supplementary Table S4.

Supplementary Table S5.

Supplementary Table S6.

Supplementary Table S7.

Supplementary Table S8.

Supplementary Table S9.

Supplementary Table S10.

## Data Availability

Compensated flow cytometry data are publicly available at https://hpap.pmacs.upenn.edu. Please contact WMH for instructions on how to download the data.
