# Supplementary material for: Multivariate indicators of disease severity in COVID-19
Source: Sci Rep. 2023 Mar 29;13:5145. doi: 10.1038/s41598-023-31683-9 (PMC10054197; doi:10.1038/s41598-023-31683-9)
Supplement: Supplementary file 1 — Supplementary Legends. [file 41598_2023_31683_MOESM1_ESM.pdf]

Figure S1. The proportion of variables exhibiting normal distribution in four datasets: Healthy controls, COVID-19 patients with moderate disease, COVID-19 with severe disease, and all COVID-19 patients (moderate and severe).

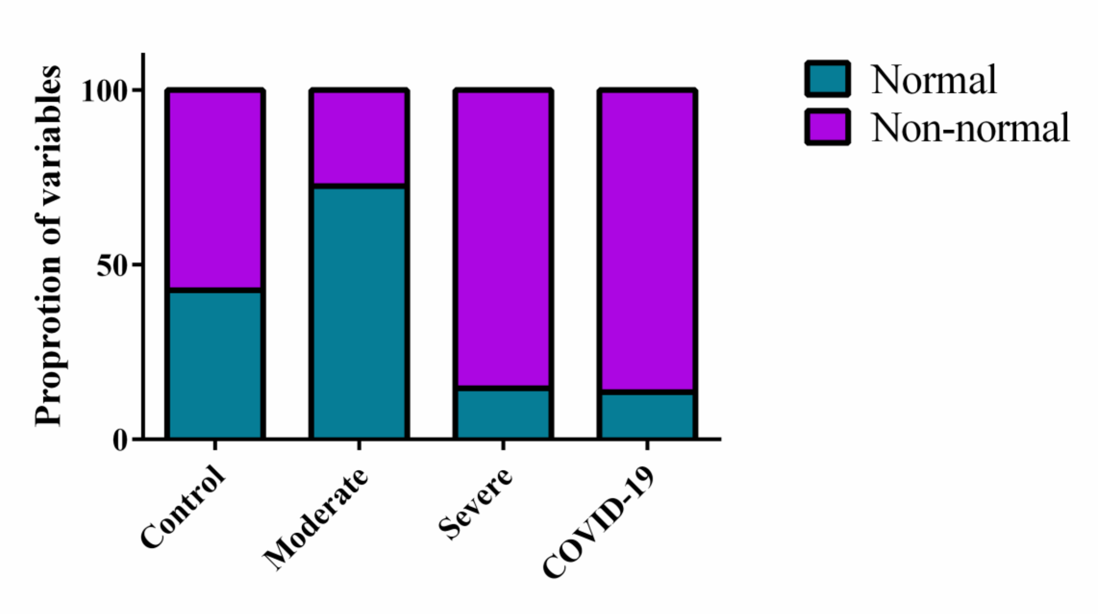

Table S1. Summary of normal distribution testing. Shapiro-Wilk statistic and *p*-values, skewness, and kurtosis data are shown for each of 171 variables in 4 datasets, healthy controls, moderate COVID-19, severe COVID-19, and combined moderate/severe COVID-19 participants. Heatmap colors indicate adherence to normal Gaussian distribution (blue) or lack of it (red).

Table S2. Multicollinearity testing. A correlation matrix was calculated for 171 variables using Pearson moment correlation coefficient for model 1.

Table S3. Multicollinearity testing. A correlation matrix was calculated for 171 variables using Pearson moment correlation coefficient for model 2.

Table S4. Multicollinearity testing. A correlation matrix was calculated for 171 variables using Pearson moment correlation coefficient for model 3.

Table S5. Homoscedasticity testing using the non-parametric Levene's test. 171 variables were tested for models 1, 2, and 3. Heteroscedasticity is highlighted in pink.

Table S6. Identification of potential outliers in the control dataset. The first and third quartiles (Q1 and Q3, respectively), as well as the lower and upper limits are shown. Potential outliers are highlighted in pink.

Table S7. Identification of potential outliers in the moderate COVID-19 dataset. The first and third quartiles (Q1 and Q3, respectively), as well as the lower and upper limits are shown. Potential outliers are highlighted in pink.

Table S8. Identification of potential outliers in the severe COVID-19 dataset. The first and third quartiles (Q1 and Q3, respectively), as well as the lower and upper limits are shown. Potential outliers are highlighted in pink.

Table S9. Identification of potential outliers in the combined (moderate/severe) COVID-19 dataset. The first and third quartiles (Q1 and Q3, respectively), as well as the lower and upper limits are shown. Potential outliers are highlighted in pink.

Table S10. Outliers in all datasets. Outliers are indicated in red without the values presented in tables S6-S9.
